# Orchids as epistemological tools: species recognition in the botany of Carolus Clusius

**DOI:** 10.1007/s40656-026-00773-y

**Published:** 2026-11-11

**Authors:** Botond Gaál

**Affiliations:** Hungarian University of Agriculture and Life Sciences, Budapest, Hungary

**Keywords:** Carolus Clusius, Early modern botany, Species ontology, Biological classification, Empirical observation, Retrospective identification

## Abstract

Early modern botanical works represent an elementary yet challenging chapter in the history of biological classification. This study re-assesses the orchid species descriptions of Carolus Clusius (1526–1609), focusing on his *Rariorum aliquot stirpium* (1583) and *Rariorum plantarum historia* (1601). Combining philological analysis with modern taxonomy, it examines how Clusius documented Central European *Orchidaceae* through empirical observation. Rather than treating retrospective identification as a teleological exercise, the study employs modern taxonomy as a diagnostic baseline to evaluate the epistemic status of Clusius’s observational categories and to determine whether they correspond to stable biological entities. Close analysis of his Latin diagnostic texts and woodcut illustrations reveals a high degree of correspondence between many pre-Linnaean categories and modern species concepts. Cases of seeming taxonomic ambiguity often reflect Clusius’s accurate representation of phenotypic plasticity and natural hybridization as distinct from classificatory confusion. The results of this research indicate that the empirical traditions of sixteenth-century botany were already predisposed to the recognition of morphologically stable natural entity independent of formal taxonomic classification. By situating Clusius’s orchid corpus within broader debates on species ontology and empirical individuation, this study contributes to ongoing discussions about the historical basis of biological classification.

## Introduction

The botanical literature of the sixteenth and seventeenth centuries was an essential transitional period between the descriptive accounts of nature written in the style of ancient authors and the modern taxonomy based on observation (Swan, 2011). Renaissance botanists introduced new methods of botanical research, such as botanical expeditions, herbaria, and field observations (Egmond, 2018). However, this process of innovation did not mean that there was a radical departure from the past, as researchers were still integrating their results with those of classical authors such as Dioscorides and Theophrastus (Elliott, 2011; Minelli, 2025). The use of Latin as the language of science had the advantage of facilitating communication but also presented the problem of "terminological instability" when early modern botanists categorized the various regional floras using an inherited terminology (Baillaud, 2001; McNeill, 1997).

*Orchidaceae* represented a particular "epistemological frontier" for Renaissance botanists; their extraordinary morphological diversity, short flowering seasons, and hidden vegetative phases pushed the boundaries of early modern identification and classification. Orchids constitute a demanding test case due to their high ecological specificity, frequent intra-specific variation, and high phenotypic variability (Dressler, 1981; Kothakota et al., 2024). Leafless, mixotrophic, or rhizomatous orchids, whose appearance differed from the "typical" morphology of orchids in early modern Europe, often gave rise to comparisons that may now look misleading today (de l’Obel, 1576; Jakubska-Busse et al., 2017). Early modern botanical classification was largely based on analogical reasoning, which established connections between the morphological features and the perceived essence of plants (de Cambiaire, 2023). Analogy served as an epistemological tool enabling naturalists to integrate diverse taxa into a coherent "order of nature" (Čermáková, 2018).

This paper focuses on Carolus Clusius' (Charles de l'Écluse, 1526–1609) descriptions of orchids. Clusius was a Renaissance humanist in the transition toward descriptive botany, specifically within the context of the Central European flora (Egmond, 2010). The selection *of Rariorum aliquot stirpium historia* (1583) and *Rariorum plantarum historia* (1601) as the primary sources for this study is justified by their central role in documenting the Pannonian^1^ landscape. Clusius published extensive botanical descriptions where he recorded morphology alongside ecology, phenology, and sensory data such as scent and taste (Clusius, 1583a, 1601). In contrast, *Stirpium nomenclator Pannonicus* (1583, 1584) is a floristic nomenclature published without illustrations (Clusius, 1583b, 1584). The persistent ambiguity in his early nomenclatural lists for instance, the taxon listed as 'Pſeudodamaſonium, Calceolus Mariæ', which has eluded identification in previous scholarship (Szabó, 1992), highlights the necessity of a comparative analysis. In this context, the cultural and linguistic reception of Clusius’ plant names in Hungary has already been documented by Barlay (1948, 1949), illustrating the deep influence of Clusian terminology on the development of Hungarian botanical language. Despite these linguistic foundations, Clusius’s orchidological records have primarily been analysed on an ad hoc basis. Past research has focused on isolated iconic taxa, leaving his broader work unexamined as a coherent observational system (Christ, 1913; Teoh, 2019). While scholarship has addressed specific cases, such as the early depictions *of Cypripedium calceolus* (Cribb, 2014) or the nomenclatural history of *Platanthera* variants (Lorenz et al., 2025) a comprehensive evaluation of his entire orchidological corpus remains absent. In some cases, modern taxonomic research specifically refers back to Clusius' records to demonstrate that his observations were often morphologically accurate, yet they operate within the distinct conceptual and linguistic paradigm of pre-Linnaean nomenclature, and as a result demand comparison with the modern taxonomic framework (Lorenz et al., 2025). Pre-Linnaean classification is complex, since the systems developed in different cultures and had different aims. While this methodological diversity makes our contemporary interpretation complicated, it also allows for a reassessment of current-day taxonomic practices by uncovering how early modern botanists recorded plant variation (Ellen, 2016; Ogilvie, 2006; Paterlini, 2007).

The ontological status of pre-Linnaean species remains contested. While Foucault (1970) has often characterized early modern botany as a primarily lexical or utilitarian enterprise, and Mayr (1982) dismissed it as rigidly 'essentialist,' my findings suggest a different perspective. The emergence of a coherent species ontology is often assumed to begin with formal binomial nomenclature. However, I examine whether stable biological entities were already recognized through empirical practice prior to theoretical codification. By juxtaposing Clusius’s holistic observational strategy with the classical Linnaean standard, I argue that his orchid descriptions offer a more refined understanding of present day scientific epistemology, and reveal a structured engagement with natural units that transcends mere nomenclature.

Here, I address a central question: how can the retrospective analysis of pre-Linnaean descriptions refine our understanding of species ontology in modern botany? By examining Clusius’s orchid corpus, I argue that his descriptions reveal a consistent effort to delineate morphologically stable units grounded in empirical observation, offering a model for more integrative botanical documentation. This work aims to reassess Carolus Clusius’ orchid descriptions by using modern taxonomy as a scientific control group to avoid a teleological reading. Such retrospective identification allows us to evaluate the structural coherence inherent in Clusius’s descriptive practices. The analysis then enable a systematic examination of Clusius’s work in order to: (1) assign selected pre-Linnaean taxa to modern species with defined confidence levels, (2) identify the diagnostic characters underlying Clusius’ classifications, and (3) demonstrate that his observations were directed toward objective biological entities, reflecting a methodological shift within the existing botanical practices toward an increased focus on the documentation of natural diversity. By explicitly examining both unambiguous and ambiguous cases, the study seeks to clarify the taxonomic interpretability of early modern botanical sources and to demonstrate their relevance for understanding historical species concepts and the early modern engagement with phenotypic variation.

## Material and methods

### Source texts and selection of chapters

Two major works by Carolus Clusius form the primary source base for examining late Renaissance botanical observation and classification. The selection of *Rariorum aliquot stirpium historia* (1583) and *Rariorum plantarum historia* (1601) as the basic sources of information is attributed to their significance for the first comprehensive botanical survey of the Pannonian and Austrian lands. The second work is considered a developed synthesis of the first one with more detailed and accurate descriptions of the morphology and habitat of plants and it contains extensive data covering the whole of Europe, as well as rich iconographic material. It is necessary to perform a comparative study of these two works in order to follow the development of his diagnostic standards and the fixation of his botanical terminology during a period of twenty years. High-resolution digital scans of the original copies were consulted to ensure a precise analysis of both the 16th-century typography and the original woodcut illustrations*. Caroli Clusii Atrebatis Rariorum aliquot stirpium per Pannoniam, Austriam et vicinas quasdam provincias observatarum historia* (Antverpiae: Christophorus Plantinus, 1583) was retrieved through the Hungarian Electronic Library (MEK), and the original book is in the National Széchényi Library, Hungary. The *Rariorum plantarum historia* (Antverpiae: Ex Officina Plantiniana, Apud Ioannem Moretum, 1601) was accessed through the Biodiversity Heritage Library, using a copy from the Digital Library of the Royal Botanical Garden of Madrid. The chapters were specifically selected because they contain morphological descriptions and accompanying illustrations that focus on modern biological entities classified in the *Orchidaceae* family.

The following chapters from the book *Caroli Clusii Atrebatis Rariorum aliquot stirpium per Pannoniam, Austriam et vicinas quasdam provincias observatarum historia* (1583) formed the basis of the research:

- *De Orchide* Cap. XXVI., 235–241
- *De Limodoro Avstriaco* Cap. XXVII., 241–242
- *De Psevdodamasonio* Cap. XXXII., 271–274
- *De Elleborine recentiorvm* Cap. XXXIII., 274–277
- *De Dentariis vvlgo dictis* Cap. VII., 444–454

The following chapters from the book *Rariorum plantarum historia* (1601) formed the basis of the research:

- *Orchides* Cap. LXI., 267–270
- *Leimodoron* Cap. LXII., 270–271
- *Elleborine recentiorvm* Cap. LXIIII., 271–275
- *Dentaria, vvlgo dicta* Cap. XXII., CXIX–CXXIIJ

This corpus enables an integrated philological and botanical analysis, whereby the Latin diagnostic text written by Clusius is cross-referenced with the visual information of the woodcuts in order to reconstruct his observation strategy and taxonomic limits.

### Philological treatment of the Latin texts

This study maintains a high degree of philological conservation to preserve Clusius’s original descriptive logic. To balance readability with philological accuracy, original orthography^2^ is preserved for plant names and headings, while long descriptive passages are adapted to modern spelling without semantic alteration. Although the original descriptive sequence is generally preserved to ensure that diagnostic morphological and ecological information remains intact, thematic reordering of specific passage elements has occasionally been employed for the sake of clarity, while ensuring that the semantic integrity and original terminology of Clusius’s text remain strictly maintained. Translations are governed by a principle of "functional fidelity," where morphological and ecological accuracy are privileged over literary translation. In the main text, translations are accompanied by their corresponding Latin texts, which represent the main evidentiary record. Latin expressions were evaluated within the broader context of Clusius’s corpus, prioritizing functional botanical description over strict lexical equivalence to bridge the gap between early modern and present day scientific terminology. Where there was ambiguity that could not be resolved, competing interpretations were maintained to represent the semiotic instability of pre-Linnaean botany.

### Treatment of pre-Linnaean nomenclature

The plant names used by Clusius were considered primarily as "descriptive markers" as opposed to fixed taxonomic units. Since Clusius's corpus predates Linnaeus' binomial nomenclature, no a priori equivalence with modern taxon names was assumed. On the contrary, historical categories such as Orchis, Elleborine, and Leimodoron were treated as fluid conceptual frameworks, evaluated through the internal evidence of Clusius’s morphological and phenological prose. Therefore, modern taxon names are used exclusively as "analytical benchmarks" to calibrate the precision of Clusius’s observations. This methodological dualism projects historical names onto a modern taxonomic grid. So as to avoid conceptual blurring, pre-Linnaean names are set in roman type, while modern botanical names are italicized. The retrospective identification undertaken here uses modern taxonomy as an epistemic probe. By testing whether his descriptive categories map consistently onto present-day biological species, the degree to which his observations captured stable natural units, as opposed to purely nominal groupings,—can be evaluated. The ontological terminology employed here is analytical, intended to clarify Clusius’s empirical structures without ascribing modern metaphysical commitments to a sixteenth-century author.

### Criteria for modern orchid identification

Modern orchid identification commonly combines vegetative and reproductive morphology with geographical and ecological data, though the diagnostic value of individual characters varies across taxonomic groups. Morphological studies of the *Orchidaceae* show that both floral and vegetative features provide taxonomically informative evidence; however, character overlap and homoplasy can limit their resolution in closely related groups (Freudenstein & Rasmussen, 1999). Recent integrative research shows that resolving species boundaries in morphologically complicated orchid groups often requires a combination of morphological, chemical, and molecular evidence (Johnson et al., 2025). Floral scent offers additional information in groups driven by specific pollinator interactions, although wide variation within and among populations limits its utility as a universal diagnostic tool (Dormont et al., 2019; Schiestl & Schlüter, 2009). Taste no longer has a meaningful role in our contemporary botanical identification, where morphological characters remain the main tool for taxon recognition. In the present research, these categories provide a comparative baseline for evaluating the information recorded by Clusius’s, including morphology, habitat, geographical distribution, phenology, and sensory observations where available.

### Morphological and ecological character analysis

For each taxon described by Clusius, all morphological, ecological, and phytogeographical data explicitly documented in the Latin text were isolated. This qualitative data was then categorized into diagnostic matrices, such as flower morphology, vegetative structures (aerial and subterranean), phenology, and habitat. These categorized properties were then cross-referenced with the standard diagnostic characters used in modern *Orchidaceae* taxonomy. For each proposed modern taxon, current distribution patterns were checked against the Euro+Med PlantBase and the database POWO (Plants of the World Online) to verify biogeographical consistency with Clusius’s recorded localities. While chorological data helped narrow down biogeographical plausibility, identifications were primarily driven by the morphological evidence preserved in Clusius’s descriptions and illustrations.

## Results

Clusius's plant classifications were primarily based on a morphological characteristics: he formed groups based on similarities in leaves, tubers, and flowers, while often referring to their relationship to other plant groups based on other morphological characteristics, such as "orchid-like" flowers or seeds. For instance, he notes the orchid-like seeds of certain species, but classifies the plant in a different group because it differs from the typical characteristics of 'orchis' at the time.

Of the plant groups recognized by Clusius, orchids in the modern sense appear in five separate groups in the 1583 edition of his work, while in the 1601 edition they appear in four groups, as one of the earlier groups was merged into another. Clusius distinguishes the individual taxa within each group by numerical designations and often delineates the main diagnostic characteristics, for example by explicitly referring to differences in leaf size.

The following analysis presents the systematic mapping of Clusius’s orchid descriptions onto modern taxonomic categories. Each entry is treated as a record of empirical observation, where the linguistic data of the Latin text is analysed in conjunction with the woodcuts as visual references within their iconographic tradition of the period. The identifications below provide case studies for examining the empirical stability of Clusius’s units of observation, through comparison with modern taxonomic categories.

### De Orchide (1583) & Orchides (1601)

Clusius highlights the principal morphological similarities of orchids in a consistent manner in both of his works. He devotes significant attention to the group of twin-tubered orchids, describing them as follows:

"*binis crassis bulbis, altero succi pleno, alterò flaccidiore, quod in orchidibus vulgare est*"

—that is, possessing two thick tubers, one filled with sap and the other more flaccid, a condition that Clusius considers typical of orchids in general.

He also includes within this group forms that differ slightly in shape, namely those with finger-like tubers:

"*Quae vero sequuntur, geminis licet radicibus sint praeditae, in orbem tornatae non sunt, sed in aliquot appendices desinunt, quasi palmas imitantur*."

—these plants likewise have two tubers, but they are not rounded; instead, they divide into several lobes, resembling the shape of a hand.

In addition, Clusius identifies the fruit and seeds as shared characteristics of orchids:

"*ut in reliquis orchidibus*, *torosum* & *contortum est capitulum exiguum semen scobis instar continens*"

—as in other orchids, the fruit is a swollen and twisted capsule containing extremely small seed, likened to sawdust.

### Orchis I

The description of the plant appears in both works, with a relatively detailed description of the vegetative parts, based on comparison as well.

*[…] Bina habet haec orchis folia […] Solum mihi conspecta est haec orchis Maio mense anni […] binis ultra Warasdinum miliaribus. […] Flores in summo spicatim cohaerentes, cuius autem coloris, cujusve formæ, ignoro, cum, ante quam aperirentur, plantam dumtaxat observaverim. Radices binas, crassas, parvi napi modo oblongas […].*

Caroli Clusii, *Rariorum plantarum historia* (Antverpiae, 1601), p. 267.

*[…]* This orchid bears two leaves. *[…]* I observed this orchid only once, in the month of May of that year *[…]*, two miles beyond Varaždin. *[…]* The flowers are clustered at the summit of the stem in a spike; however, of what colour or form they are I do not know, since I observed the plant only before the flowers had opened. It has two thick roots, elongated in the manner of a small carrot *[…]*.

In the margin of the page, Clusius explicitly draws attention to “*latifol. max.*” in 1583 and “*ampliss. folijs*” in 1601, referring to the unusually broad leaves. The description is compatible with the genus *Platanthera Rich.*, most notably *P. bifolia* (L.) Rich. and *P. chlorantha* (Custer) Rchb., with the latter providing the closest overall match. Species lacking tubers (e.g. *Cephalanthera* Rich., *Epipactis* Zinn) or with rounded tubers (e.g. *Orchis* Tourn. ex L., *Neotinea* Rchb.f.) can be excluded. Clusius’s ability to provide a taxonomically interpretable record even in the absence of floral data demonstrates that his observational units were grounded in a holistic structural understanding.

### Orchis latifolia altera

**Fig. 1:**
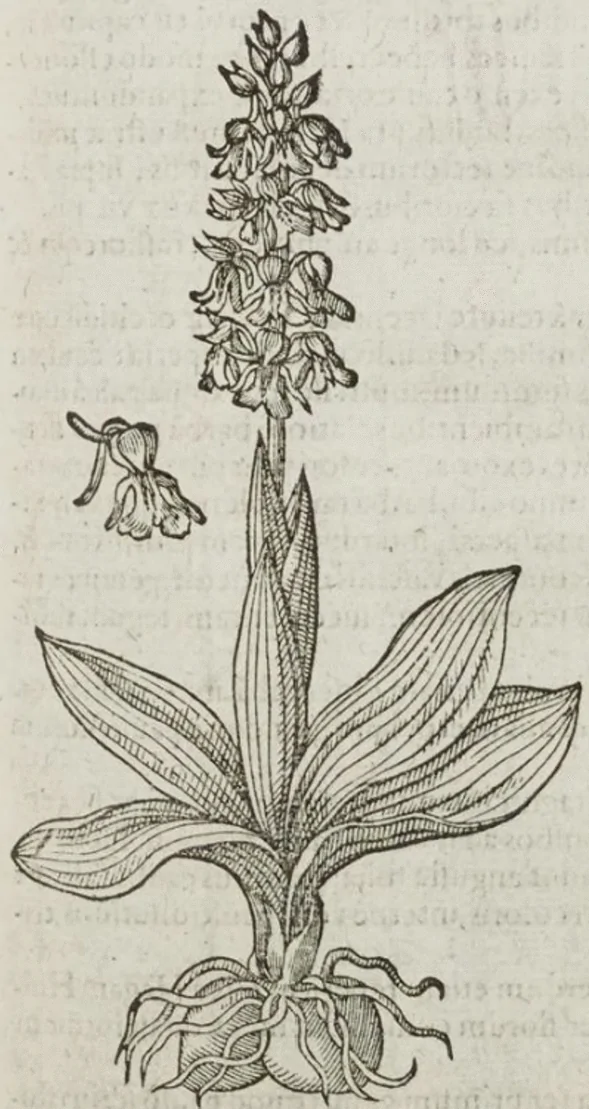
Orchis latifolia altera Caroli Clusii, *Rariorum plantarum historia* (Antverpiae, 1601), p. 267.

In none of his books does he treat it as a completely separate plant compared to the previous one. It was not assigned a distinct number. Clusius describes the characteristics of the plant in detail in both of his works, and also includes an iconographic illustration in the edition of 1601 (Fig. 1).

*[…] Foliis quidem magnis & latis, sed infra superiorum amplitudinem, caule propemodum cubitali, floribus magnis, aliarum orchidum forma purpureis maculis aspersis. […] Radicibus geminis, crassis, minus tamen superiore oblongis. […] Observavi non solum in silvis Nemethwywar, […] sed etiam in silva Leytana super Wanderstorf.*

Caroli Clusii Atrebatis Rariorum aliquot stirpium per Pannoniam, Austriam et vicinas quasdam provincias observatarum historia (1583), p. 237.

*[…]* The leaves are large and broad, but smaller than those of the previous plant; the stem is nearly cubital in length; the flowers are large, showing the form typical of other orchids, and are sprinkled with purple spots. *[…]* The roots are paired and thick, but less elongated than those of the former species. *[…]* I observed this orchid not only in the forests near Nemethwywar^3^*[…]* but also in the Leytana forest above Wanderstorf.

The described morphology and accompanying illustration most plausibly associate the plant with *Orchis mascula* L.. Other *Orchis* species cannot be excluded based on the text. *Orchis purpurea* Huds. also has purple-spotted flowers. By treating this plant as a subordinate variant rather than a numbered species, Clusius acknowledges a degree of morphological continuity within the Orchis group.

### Orchis II

The description of this plant is relatively brief in both works and there may be several possible options.

*Plura sunt folia, angustiora, caulis gracilior, pedalis, flores rubri pulcherrimi*. *[…]* *Quod e pratis eruebatur*. *Crescit iugo Grebén*, & *plerisque gramineis locis*, *inter flumina Muram* & *Dravm* *[…]*.

Caroli Clusii *Atrebatis Rariorum aliquot stirpium per Pannoniam, Austriam et vicinas quasdam provincias observatarum historia* (1583), pp. 237–238.

It has more leaves, narrower, a slenderer stem, a foot-long stem, and the most beautiful red flowers. It was dug up from the meadows. *[…]* It grows on the ridge of Grebén and in most grassy places between the rivers Muram and Drau. *[…].*

Several taxa of *Gymnadenia* R.Br.*, Anacamptis* Rich. and *Orchis s.l.* may correspond to this description; however, the available information does not allow a reliable species-level identification. The persistent ambiguity of this record highlights the "epistemic limitations" of early modern botanical prose, where the lack of technical vocabulary describing the microstructure of flowers prevents the definitive separation of morphologically similar genera, which modern taxonomy distinguishes on the basis of minute reproductive details.

### Orchis III

A similarly brief description is provided; however, Clusius mention the fragrant flowers. This is a diagnostic feature that excludes a number of otherwise habitually similar taxa.

*[…] Binis crassis bulbis preditum, uno succi pleno, altero flaccidiore[…]. Foliis longis, angustis: caule interdum pedali, spica florum elegante, odorata. Quorum galeola obscura purpura nitet, habitus cum propendente labro candicant, purpureisque maculis insigniuntur. Invenitur in pratis Nemethwywar […].*

Caroli Clusii, *Rariorum plantarum historia* (Antverpiae, 1601), p. 267.

*[…]* It has two thick tubers, one full and the other more flaccid *[…]*. Its leaves are long and narrow; the stem is sometimes a foot long, and the flower spike is elegant and fragrant. The flowers’ helmet shines in dark purple, the pendent labellum is whitish, and they are marked with purple spots. It is found in the meadows near Nemethwywar *[…]*.

The combination of twin tubers, narrow leaves, fragrant flowers, and a dark-coloured helmet places the plant within the *Neotinea* group. Among the European species, the described morphology most closely matches *Neotinea ustulata* (L.) R.M.Bateman, Pridgeon & M.W.Chase, although related taxa cannot be fully excluded based on the historical description alone. The prioritization of scent data and morphology indicates that Clusius' empirical method relied on multiple senses rather than just visual characteristics, but purely structural descriptions can remain ambiguous.

### Orchis IIII

**Fig. 2:**
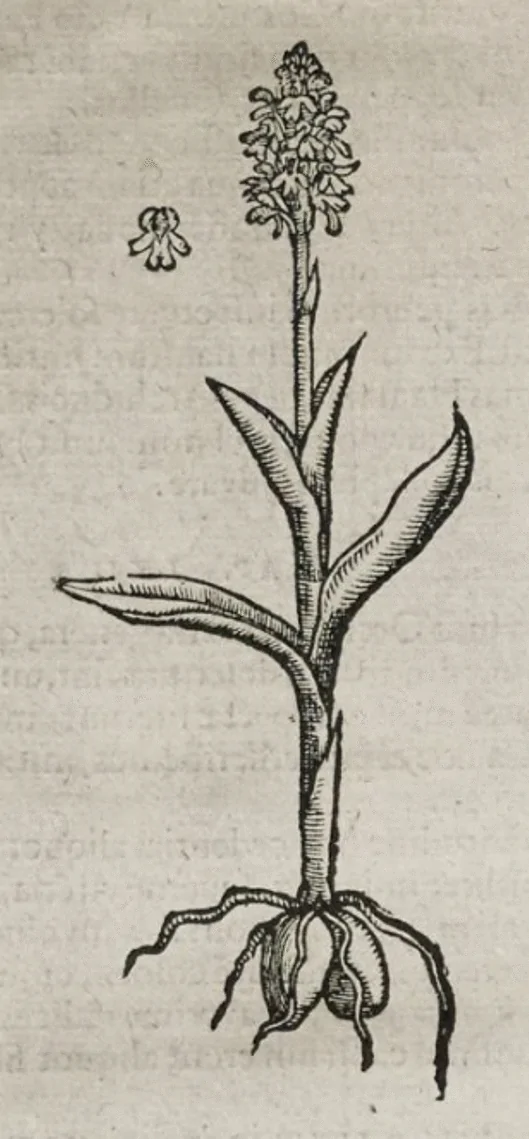
Orchis Pannonica IIII. Caroli Clusii *Atrebatis Rariorum aliquot stirpium per Pannoniam, Austriam et vicinas quasdam provincias observatarum historia* (1583), p. 236.

4Clusius describes the plant in detail in both of his works, and both books include illustrations of the plant (Fig. 2). He also refers to the plant as Pannonica (Orchis Pannonica), indicating where he found it.

*Qliuibus. […].lcari a tergo prominente, tenui odore. […] Geminas radices habet parvorum testiculorum modo. […] Copiose admodum in montium tenui gramine tectorum accuina aut sena sunt folia, caulis palmaris aut amplior, aliquot minoribus foliis involutus. Spica elegantium minutorum florum purpurascente galea tectorum, labro prominulo, in quatuor barbulas diviso, candido, purpureisque punctis asperso. Parvo calcari a tergo prominente, tenui odore. […] Geminas radices habet parvorum testiculorum modo. […] Copioseadmodum in montium tenui gramine tectorum accliuibus. […].*

Caroli Clusii *Atrebatis Rariorum aliquot stirpium per Pannoniam*, *Austriam et vicinas quasdam provincias observatarum historia* (1583), p. 238.

It has five or six leaves; the stem is about a hand’s length or longer, surrounded by a few smaller leaves. Its inflorescence consists of small, elegant flowers covered by a purplish hood; the lip is projecting, divided into four small lobes, white with purple spots. A small spur projects from the back, and the flowers are faintly scented. *[…]* It has two roots, shaped-like small testicles. *[…]* It is very abundant on the sparsely grassy slopes of the mountains. *[…].*

Based on the described morphology, the plant can be assigned to the genus *Neotinea*. The combination of small, elegant flowers with a purplish helmet, a projecting white labellum with purple spots, a short spur, faint fragrance, and twin tubers is provides a compelling match for *Neotinea tridentata* (Scop.) R.M.Bateman, Pridgeon & M.W.Chase*.*The description of the labellum as divided into four small lobes falls within the morphological variation observed in this species. The illustration, however, clearly resembles *Neotinea ustulata*. For this reason, the taxon cannot be ruled out as a potential match for Clusius's record. The similarity of the illustrated plant to *N. ustulata* raises the possibility that Clusius observed intermediate forms, potentially *N.* × *dietrichiana* (Bogenh.) H.Kretzschmar, Eccarius & H.Dietr.*.* The discrepancy between Clusius's text and illustrations in this case suggests that his notes functioned more as cumulative observations of populations than as snapshots of individual specimens.

### Orchis V

**Fig. 3:**
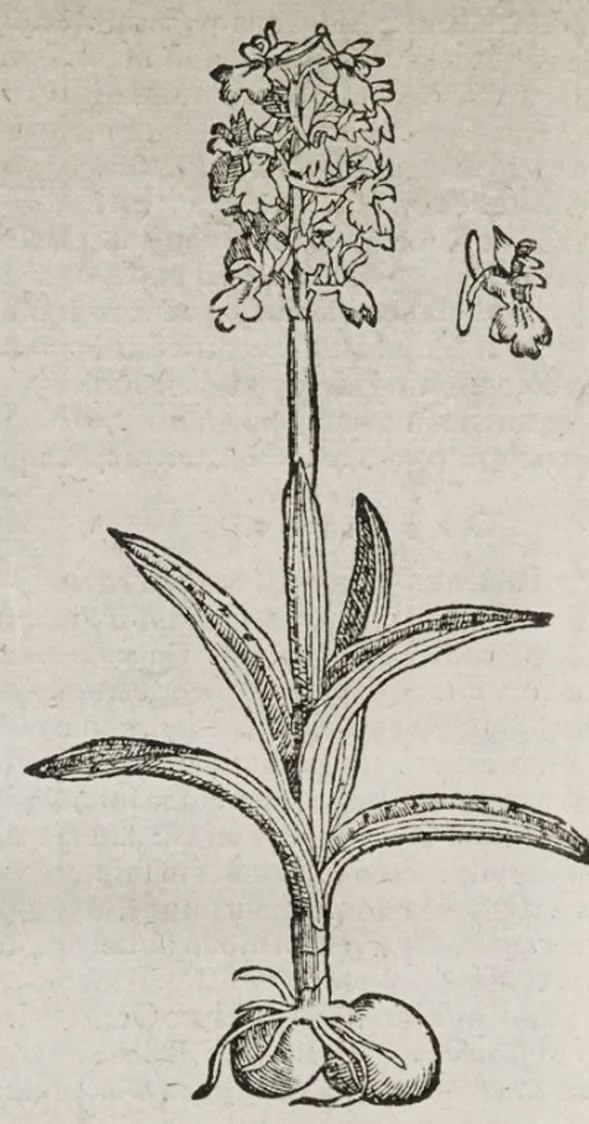
Orchis V. Caroli Clusii, *Rariorum plantarum historia* (Antverpiae, 1601), p. 268.

Clusius describes the plant’s morphology in detail, noting its resemblance to a Low Countries orchid, and explicitly states that he observed similar specimens in the wild near Vienna and in the vicinity of London. His book, published in 1601, also includes an illustration of the plant (Fig. 3).

*Quatuor aut plura habet folia, summa tellure iacentia. […] Caulem palmarem, aliquot minoribus foliis septum. […] latiore barba propendente, & obtusiore calcari praediti […]. Coloris aut purpurei saturioris, aut dilutioris, aut fere carnei, atque etiam omnino albi. Barba tamen plerumque vel exiguis, vel maioribus maculis sature purpurascentibus aspersa, interdum etiam prorsus nullis, praesertim in lacteis floribus. […] Geminas radices, unam superioris anni flaccidiorem. […].*

Caroli Clusii, *Rariorum plantarum historia* (Antverpiae, 1601), p. 268.

It has four or more leaves lying flat on the surface of the ground. *[…]* The stem is of a hand’s length, surrounded by a few smaller leaves. […] It is furnished with a broader, pendent lip and a blunter spur *[…]*. Its colour may be a deeper or paler purple, almost flesh-coloured, or even entirely white. The lip, however, is usually sprinkled with smaller or larger dark purple spots, though sometimes completely without spots, especially in milky-white flowers. *[…]* It has two tubers, one of which belongs to the previous year and is more flaccid *[…]*.

According to the described morphological characteristics, the highly variable flower colour variations and woodcut, it can be identified as *Anacamptis morio* (L.) R.M.Bateman, Pridgeon & M.W.Chase. Clusius’s recording of the range of flower colours within a single population suggests that he treated such variation as part of the observed plant rather than as sufficient grounds for distinguishing separate taxa. This approach reflects an empirical focus on documenting natural variation, which allows us to view his descriptions as accurate records of phenotypic plasticity, regardless of the absence of modern theoretical frameworks.

### Orchis VI

**Fig. 4:**
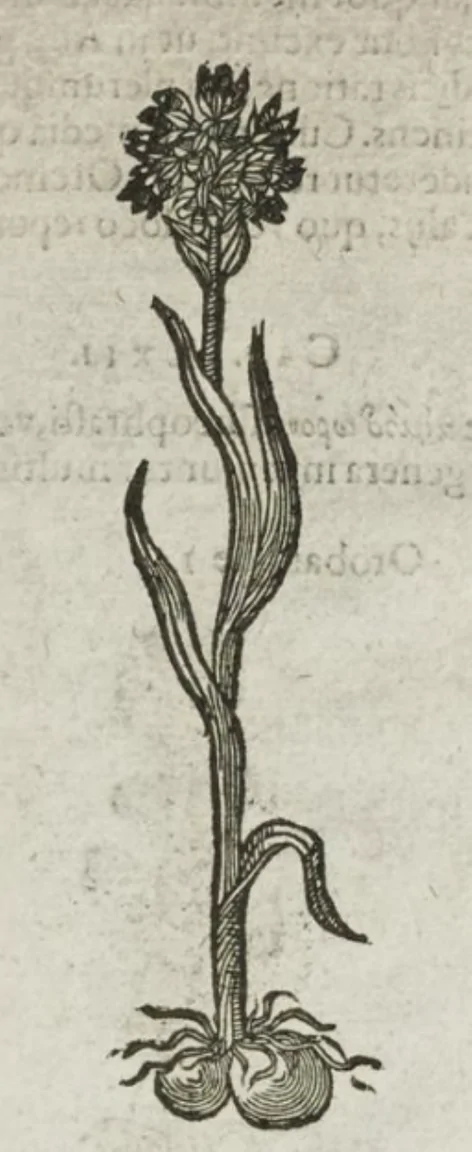
Orchis VI. (Orchis Batavica). Caroli Clusii, *Rariorum plantarum historia* (Antverpiae, 1601), p. 269.

This plant is illustrated (Fig. 4) and described by Clusius in the 1601 edition and not present in the 1583 edition. Therefore, the orchid marked Orchis VI. is not the same as the one in the 1583 edition; from this point on, the numbering shifts.

*Ingrati etiam odoris est. […] Dodrantali assurgit caule, quem terna aut quaterna ambiunt angusta folia. Summus caulis sustinet breviorem spicam florum elegantis interius rubri coloris, interne vero paulo dilutioris, trifido labro propendente. […] Cuius iconem & binos bulbos ad me misit ante biennium [Ioan. Hoghelandius]. […].*

Caroli Clusii, *Rariorum plantarum historia* (Antverpiae, 1601), p. 268

It is also of unpleasant odor. *[…]* It rises on a three-quarter foot stem, which is surrounded by three or four narrow leaves. The upper part of the stem bears a short inflorescence of elegant flowers, red in colour on the inside, though slightly paler internally, with a projecting, three-lobed lip. *[…]* Johannes Hoghelandius sent me its illustration and two bulbs two years ago. *[…].*

Based on the described characters, the plant is most plausibly referable to *Anacamptis pyramidalis* (L.) Rich.; since the presence of twin tubers, a slender stem bearing a compact inflorescence, and flowers with a projecting, three-lobed labellum are consistent with this species. The illustration further supports this interpretation by showing a dense and regular arrangement of the flowers.

Clusius refers to this orchid as No. VI. and as Orchis Batavica, reflecting its provenance from the Netherlands as indicated by the material sent by Johannes Hoghelandius. The records of materials sent by Hoghelandius provide evidence of the transnational networks through which Clusius received plant material and observational information, including illustrations and sensory data such as smell.

### Orchis VII. (1601) and Orchis VI. (1583)

This plant appears in both editions. Clusius describes the plant accurately, highlighting its main morphological characteristics.

*Tria plerumque habet septimum genus angusta folia […]. Cauliculum palmarem, spica flosculorum ex herbaceo pallidi coloris, tribus apiculis constantium, & moschi odorem referentium, exornatum. Unicam radicem, rotundam pisi magnitudine, supra quam longae propagines exporriguntur, quae novas subinde plantas generant. […] Iunio floret.*

Caroli Clusii, *Rariorum plantarum historia* (Antverpiae, 1601), p. 269

The seventh genus^5^ usually has three narrow leaves […]. It produces a hand-length stem, with an inflorescence of small, pale greenish flowers, each with three points and a musky scent. It has a single, round tuber about the size of a pea, above which long shoots develop, periodically generating new plants. […] It flowers in June.

Clusius’s observation of the "long shoots" (stolons) and the single tuber demonstrates a transition from purely floral-based identification to a comprehensive life-history approach, capturing the unique reproductive strategy that remains a diagnostic hallmark of *Herminium monorchis* (L.) R.Br., which provides the best overall match.

### Orchis VIII. (1601) and Orchis VII. (1583)

**Fig. 5:**
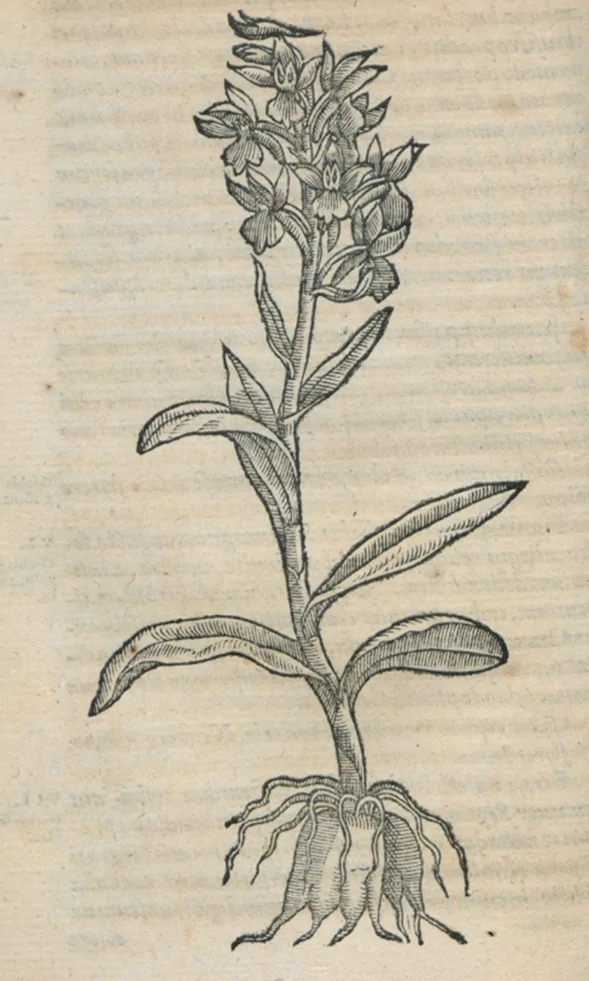
Orchis VII. Caroli Clusii *Atrebatis Rariorum aliquot stirpium per Pannoniam, Austriam et vicinas quasdam provincias observatarum historia* (1583), p. 240.

The following plant differs from those described so far, Clusius notes, as its tuber is not round. Nevertheless, it is described as an orchid. Both of his works include illustrations of it (Fig. 5). It can be identified with the help of morphological and pictorial representations.

*[…] Tribus aut quatuor foliis. […] Caulem dodrantalem amplectuntur nonnulla foliola. In cuius summo flores spicatim digesti nascuntur colore exalbido, nec ullis punctis distincto. […] Graviter odorati, florum sambuci odorem aemulantes. […] Radices geminae, veluti palmae in binos aut plures digitos distributae sunt. […] Aliud est cum forma tum magnitudine illi simile, cuius flores purpurei sunt. […] Floret autem Aprili, nonnunquam etiam Maio.*

Caroli Clusii *Atrebatis Rariorum aliquot stirpium per Pannoniam, Austriam et vicinas quasdam provincias observatarum historia* (1583), p. 239–241

*[…]* It has three or four leaves. *[…]* Several small leaves clasp the three-quarter foot length stem. At its summit, the flowers are arranged in a spike, whitish in colour and lacking any spots. *[…]* They are strongly scented, resembling the fragrance of elderflowers. *[…]* The roots are paired and divided in a palm-like manner into two or more lobes. *[…]* There is another plant similar to this one in both form and size, but with purple flowers. *[…]* It flowers in April, sometimes also in May.

Based on the described morphology, like the finger-like branching tubers and early flowering period, the plant can be identified as *Dactylorhiza sambucina* (L.) Soó. Clusius grouped the different flower coloured individuals into a single entry based on their common elderflower scent and root structure, effectively emphasizing stable structural and sensory characteristics over striking flower colour. This suggests an early recognition of species constancy, recognizing that phenotypic colour variation does not necessarily imply a taxonomic break.

### Pſeudoorchis

In the 1601 edition, in the chapter on *Orchides* Cap. LXI., Clusius describes another plant, which calls *Pſeudoorchis*. Based on morphological characteristics, he is not certain about the identification of the plant.

*Habet illa unicum folium*. *[…]* *Multis venis secundum longitudinem excurrentibus*, & *medio nervo praeditum*. *[…]* *a medio ad summum fastigium flosculis quibusdam onustus*, *quorum forma mihi ignota, quia siccam plantam duntaxat videre contigit*. *In qua inhaerebant adhuc flosculorum petioli,* & *bina aut terna capitella, instar capitulorum orchidis*. *Radix bulbosa est, aliquot membranaceis involucris tecta, praeter orchidum naturam* *[…]*. *Cum porro mediam quandam naturam habeat haec planta*, & *peculiare caput non videretur requirere*, *orchidibus* *subiiciendam esse existimavi*. *[…]*.

Caroli Clusii, *Rariorum plantarum historia* (Antverpiae, 1601), p. 269–270

It has a single leaf, *[…]* furnished with many veins running longitudinally and with a distinct midrib. […] From the middle to the uppermost part, the stem bears several small flowers, the form of which is unknown to me, since I had the opportunity to see the plant only in a dried state. On the specimen there still remained the pedicels of the flowers, as well as two or three small heads, resembling the capsules of orchids. The root is bulbous, covered by several membranous sheaths, which is contrary to the usual nature of orchids. *[…]* Since this plant appears to possess a certain intermediate nature and does not seem to require a separate chapter, I considered it appropriate to place it among the orchids*. […].*

Clusius likely placed the plant among orchids based on its floral morphology, but the vegetative organs differ. The storage organ’s bulb-like structure is a strong argument against it. The plant is more likely a monocotyledon belonging to the *Asparagaceae s.l.* family. The specimen described by Clusius cannot be linked to the genus *Pseudorchis* Ség. as we know it today. This classification reveals an early modern strategy of "taxonomic clustering," where atypical specimens were grouped with established families based on partial morphological analogies.

### De Limodoro Avstriaco (1583) & Leimodoron (1601)

The chapter entitled De Limodoro Austriaco (1583) still uses the term Limodoro in a distinctly regional and empirical manner. The term does not indicate an abstract taxonomic unit, but instead refers to a specific plant observed in the Alpen region around Baden, known as the "Austrian Limodoro." In this chapter, Limodoro refers to a heterogeneous group of plants that includes an orchid corresponding to *Limodorum abortivum* (L.) Sw. (*Orchidaceae*), several species of *Orobanche* L. (such as *O. rapum-genistae*), and Radix cava (now *Corydalis cava (L.) Schweigg. & Körte), (Papaveraceae)*, reflecting the early Clusius-style grouping based on morphological and ecobiological similarities.

By contrast, the chapter on Leimodoron in *Rariorum Plantarum Historia* (1601) represents a synthesizing and philologically grounded approach. Here, Clusius consciously returns to the Greek origin of the name and places the concept of Limodorum within the interpretative framework of the plant group described by ancient authors (Theophrastus, Dioscorides, Pliny), departing from the earlier, narrowly regional interpretation. Clusius classifies plants belonging to the Leimodoron group in a more conscious manner than before, but in a similar way, distinguishing three main units: The first unit consists of the plant discussed under the name "Leimodoron" or "Orchis abortiva," the second unit consists of the group discussed under the concept of "Orobanche legitima," which corresponds to the *Orobanche s.l.* circle in today's sense, and the third unit consists of Radix cava, similar to the 1583 edition.

### Limodoro Austriaco, Pſeudo-leimodωron

Although the two names refer to the same plant, he used the former in his work published in 1583, while the latter appeared in his 1601 work. The term ‘limodoron’ may have referred primarily to the *Orobanche* species used in modern taxonomy, which is why it was given the name Pſeudo [Pseudo].Clusius notes that the origin of the name comes from Theophrastus (c. 371–c. 287 BC), who referred to plants with a parasitic lifestyle as λειμόδωρον (leimodoron) or αἱμόδωρον (haimodoron). He described the orchid, classified in the group in today's sense, as follows:

*Orchidibus quodammodo cognatum. […] Pedali assurgit illa caule […], spicam gestans florum quatuor foliis constantium […]. Floribus striata & longa subsunt capitula, semine exili & orchidum simili plena. Subrotundam radicem non habet […], sed crassas, longas, & obliquas fibras habet […]. Tota stirps saturo purpuræ & plane violaceo colore prædita est. Floret Iunio. Similem fere, sed multo dilutioris coloris olim observare memini non modo in silva illa agri Mompelliani, in qua coenobium Gramont dictum, sub ilicis arboribus, sed aliis plerisque silvis. Non dubium est quin his adnumeranda sit, ea quam Lobelius Orchidem abortivam […].*

Caroli Clusii, *Rariorum plantarum historia* (Antverpiae, 1601), p. 270

Somewhat related to orchids. *[…]* It rises to about a foot in height on its stem *[…]*, bearing a spike of flowers each with four petals *[…]*. Beneath the flowers are striped and elongated capsules, filled with slender, orchid-like seeds. It does not have a nearly round root *[…]*, but has thick, long, and oblique fibers *[…]*. The entire plant is endowed with a deep purple and clearly violet colour. It blooms in June. I remember having observed a very similar plant, though of much paler colour, not only in that forest of the Montpellier^6^ region, where the Gramont monastery stands, under the evergreen oaks (*Quercus ilex*), but I have also seen in many other forests. There is no doubt that it should be included among these, namely the one that Lobelius called Orchidem abortivam *[…].*

**Fig. 6:**
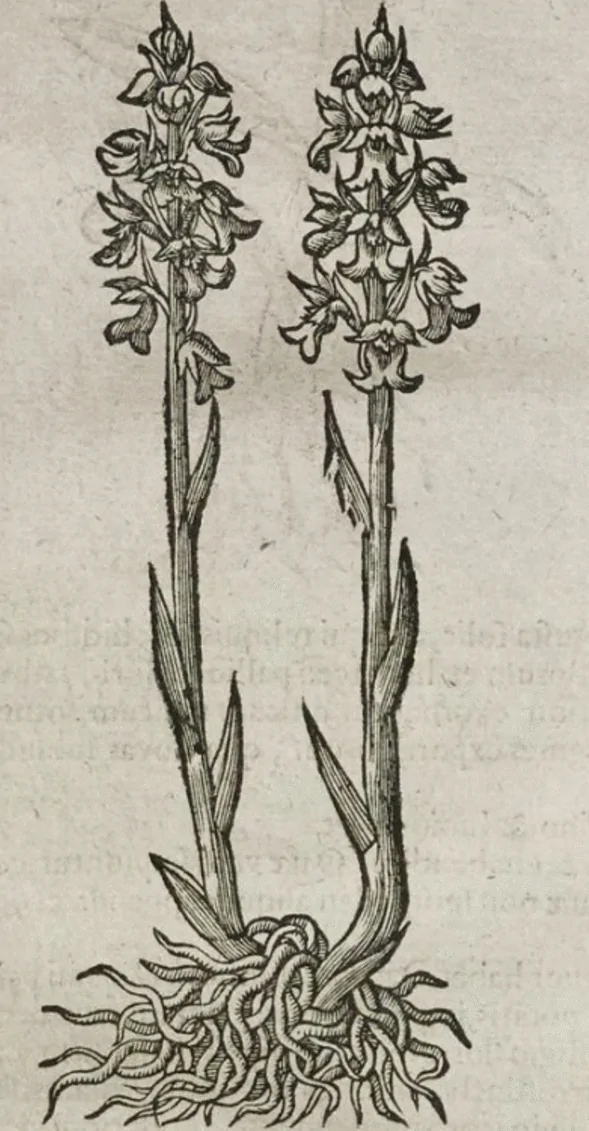
Pſeudo-leimodωron Caroli Clusii, *Rariorum plantarum historia* (Antverpiae, 1601), p. 270.

The detailed morphological description and the accompanying woodcut (Fig. 6) identify the plant as *Limodorum abortivum*. Clusius’s use of the prefix Pseudo- and his reference to Theophrastus’s leimodoron (used for parasitic *Orobanche* species) reveals a functional rather than purely phylogenetic approach to classification. By grouping this orchid with other "parasitic-looking" plants based on its violet, leaf-less habit and fibrous root system, Clusius demonstrates an ecological reasoning that prioritized life-strategy and habitus over the presence of typical orchid tubers. This case exemplifies the early modern attempt to reconcile classical botanical terminology with empirical observations of non-autotrophic growth forms.

Clusius also reports a similar plant of much paler colour observed in Mediterranean forests, which he does not separate taxonomically from the typical form but explicitly associates with the same entity, including the taxon referred to by Lobelius as “Orchidem abortivam”. This case illustrates how Clusius gradually differentiated ecological and morphological concepts that later became taxonomically distinct lineages.

### De Psevdodamasonio (1583)

This chapter appears only in *Caroli Clusii Atrebatis Rariorum aliquot stirpium per Pannoniam, Austriam et vicinas quasdam provincias observatarum historia* (1583); in the book *Rariorum plantarum historia* (1601), Clusius describes the plant mentioned in this chapter in the chapter ‘*Elleborine recentiorvm’*. Since, based on current taxonomic classification, the species described in this chapter is strongly distinct from the Elleborine group, it will be discussed separately in this paper.

*[…] Pedali aut maiore amplitudine, quem quaterna aut quina ambiunt folia. […] Ex foliis quod extremo cauli insidet, quodque reliquis minus est, nonnunquam & ex proximi sinu prodit flos unicus, oblongo pediculo inhaerens. Quatuor foliis crucis forma decussatis, oblongis et mucronatis, colore ex purpura nigricante constans. Quorum superius & inferius maiuscula sunt, lateralia vero admodum angusta, & interiore parte lanuginosa. Ex quorum umbilico membraneus quidam & tumens, intusque vacuus utriculus protuberat ovi columbini magnitudine. Superiore parte secundum umbilicum non nihil apertus & hians, instar calceoli ore patulo. Colore nunc luteo, nunc pallido, intus nonnihil villosus. […] Siliqua illi succedens oblongior est minimi digiti crassitie, triangula. Semen tenue & veluti pulvereum continens, quemadmodum orchides […]. Radix nigra est, non admodum crassa, in longum & obliquum summa tellure sese spargens. […] Floret Maio. Sunt qui dicant flore albo inveniri. […] ab incolis Marien schuch appellari.*

Caroli Clusii *Atrebatis Rariorum aliquot stirpium per Pannoniam, Austriam et vicinas quasdam provincias observatarum histori*a (1583), p. 271–274

*[…]* Of a stature one foot or greater, surrounded by four or five leaves. *[…]* From the leaf seated at the extremity of the stem, which is smaller than the others, and sometimes also from the axil of the adjacent leaf, a single flower emerges, attached to an elongated pedicel. It consists of four leaves arranged crosswise in the form of a cross, oblong and mucronate, of a dark, blackish-purple colour. Of these, the upper and lower are somewhat larger, whereas the lateral ones are very narrow and downy on their inner surface. From the centre of these a certain membranous, swollen, and internally hollow sac protrudes, of the size of a pigeon’s egg. In its upper part, along the centre, it is somewhat open and gaping, like a slipper with an open mouth. Its colour is sometimes yellow, sometimes pale, and its interior is slightly hairy. *[…]* The capsule that follows is rather elongate, about the thickness of the little finger, triangular. It contains fine, almost powder-like seeds, as in orchids *[…]*. The root is black, not very thick, spreading lengthwise and obliquely within the upper layer of the soil. […] It flowers in May. Some say it is also found with white flowers. […] It is called ‘Marien schuch’ (Marias’ slipper) by the inhabitants.

**Fig. 7:**
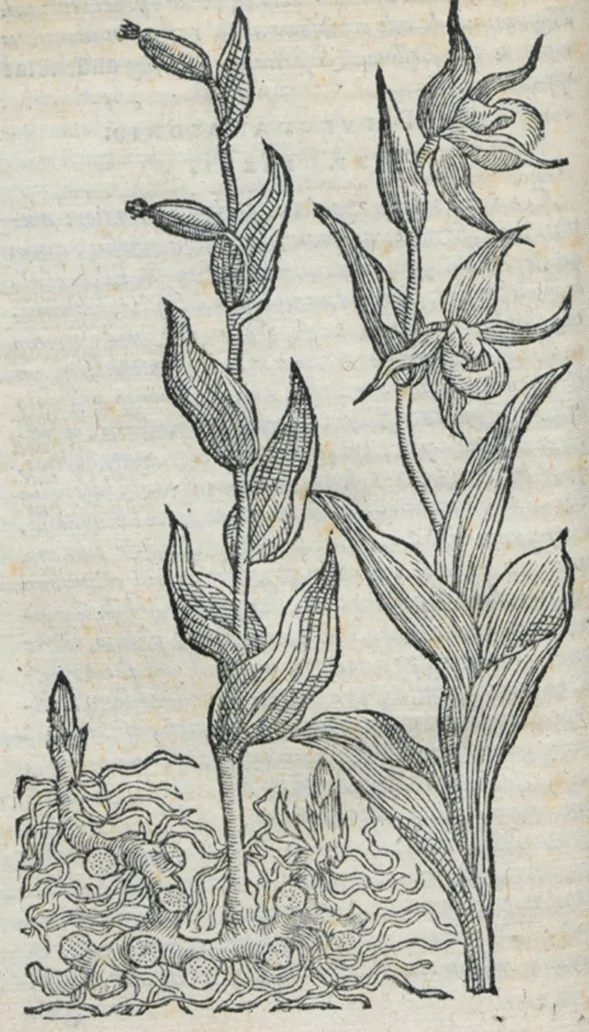
Psevdodamasonio. Caroli Clusii *Atrebatis Rariorum aliquot stirpium per Pannoniam, Austriam et vicinas quasdam provincias observatarum historia* (1583), p. 272.

Clusius’ detailed morphological description refers unambiguously to a slipper-shaped flower, a diagnostic character of the genus *Cypripedium* L.. The transversely arranged perianth segments and the membranous, swollen, hollow structure described as a “little shoe” correspond closely to the floral morphology of *Cypripedium calceolus* L.. The accompanying illustration (Fig. 7) further supports this identification.

By explicitly noting the fine, dust-like seeds typical of orchids, Clusius conceptually linked the plant to the historical orchid group. He used effectively the reproductive traits to bridge his taxonomic doubts. This indicates that despite his doubts about the plant's classification, he used the reproductive characteristics as an anchor to assign it to the orchids. In his later work (1601), however, he qualifies this relationship, describing the species as only superficially related to orchids and notes that some authors of the sixteenth century refer to it as Elleborine.

*Superioribus orchidibus congener est ea planta quæ a recentioribus nonnullis elleborine cognominatur.*

Caroli Clusii, *Rariorum plantarum historia* (Antverpiae, 1601), p. 272

Related to the aforementioned orchids is that plant which by some more recent authors is named elleborine.

The description also records colour variation, including reports of white-flowered individuals. Clusius’s recording of the vernacular name "Marien schuch" (Mary’s slipper) demonstrates his integration of local folk-botanical knowledge into a formal descriptive framework.

The orchid listed as “Pſeudodamaſonium, Calceolus Mariæ” in the *Stirpium nomenclator Pannonicus* (Clusius, 1583b, 1584) can be unambiguously identified based on the detailed description and illustration in *Rariorum aliquot stirpium per Pannoniam, Austriam et vicinas quasdam provincias observatarum historia* (1583) as *Cypripedium calceolus* L..

### De Elleborine recentiorvm (1583) & Elleborine recentiorvm (1601)

In both books, the chapter mostly contains species belonging to the present-day *Orchidaceae* family, which are presented individually in this publication. After that, both books describe the plant “Elleborus albus”, which has been identified as *Veratrum album* (*Melanthiaceae*) based on the woodcut. This plant is a standard for Clusius, who often compares other species to it, and it forms the basis of the Elleborus group based on leaf morphology. Moreover, in *Rariorum plantarum historia* (1601), are described other plants that today correspond to the *Helleborus* L. (*Ranunculaceae*) genus. In the book of *Caroli Clusii's Atrebatis Rariorum aliquot stirpium per Pannoniam, Austriam et vicinas quasdam provincias observatarum historia* (1583) classifies Psevdodamasonio (*Cypripedium calceolus)* in a separate chapter, while *Rariorum plantarum historia* (1601), includes it in this chapter, resulting in a discrepancy in the numbering of the plants described.

### Elleborine I. (1583), Elleborine II. (1601)

The descriptions of the plants in the two books are almost identical.

*Caulem habet pedalem, aut ampliorem, firmum, quem per intervalla involvunt inordinata folia sena aut septena. […] Flores in summo caule denos aut duodenos, spicatim congestos, albos. Quinis foliis constantes, & in medio veluti parvam galeam complexos subluteo labro. Non multum ab orchidis floribus distantes &, ut illi, siliquae angulosae striatae & contortae inhaerentes, inodoros. Semen in siliquis minutum scobis instar. Radices crassiusculas […] & sparsas. […] Hoc genus praecocius est reliquis, & Maio mense plerumque florere solet.*

Caroli Clusii *Atrebatis Rariorum aliquot stirpium per Pannoniam, Austriam et vicinas quasdam provincias observatarum historia* (1583), pp. 274–275.

It has a stem a foot long or more, firm, which is surrounded at intervals by six or seven irregularly arranged leaves. At the top of the stem, ten or twelve white flowers are densely clustered in a spike. *[…]* The flowers consist of five petals, enclosing in the center a small hood-like structure with a pale yellow lip. They do not differ much from orchid flowers, and like them, the attached capsules are angular, striated, twisted, and scentless. The seeds inside the capsules are tiny, like sawdust. The roots are somewhat thick *[…]* and spreading. *[…]* This genus flowers earlier than the others and usually blooms in May.

The morphological characteristics described are typical of the genus *Cephalanthera* and are consistent with the known characteristics of both *C. damasonium* (Mill.) Druce and *C. longifolia* (L.) Fritsch. The denser inflorescence may suggest *C. damasonium*, but due to morphological variability, *C. longifolia* cannot be ruled out. Clusius’s observation of the “tiny seeds” serves as his primary justification for maintaining the botanical link to orchids. This entry illustrates Clusius’s reliance on "cryptic" characters—traits not immediately visible to the casual observer—to establish taxonomic affinity.

### Elleborine II. (1583) & Elleborine III. (1601)

**Fig. 8:**
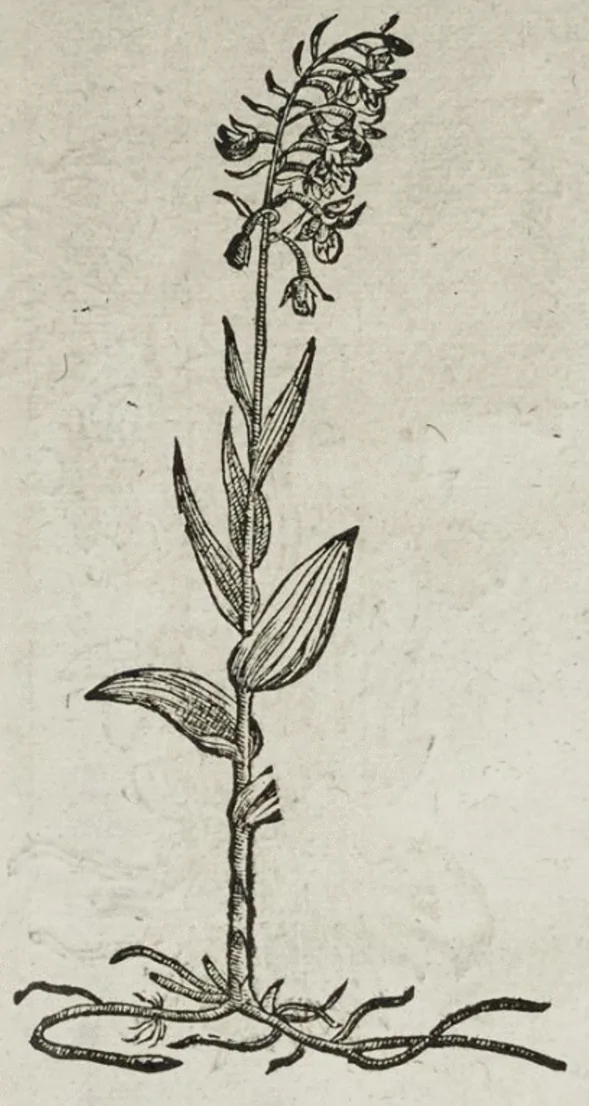
Elleborine III Caroli Clusii, *Rariorum plantarum historia* (Antverpiae, 1601), p. 273.

A brief description of the plant can be found in both publications. However, the accompanying woodcut (Fig. 8) does not appear in the 1583 edition, whereas it is included in the *Rariorum plantarum historia* (1601).

*Pari est secundæ magnitudine, paulo latioribus foliis, minoribus floribus […]. Galeolam cum prominente labro amplectuntur, colorisque sunt herbacei, interius nonnihil candicantis, nullius odoris[…]. Invenitur in quibusdam Pannoniae & Austriae siccioribus pratis […].*

Caroli Clusii, *Rariorum plantarum historia* (Antverpiae, 1601), p. 273

It is equal in size to the second, with slightly broader leaves and smaller flowers *[…]*. The flowers enclose a little hood with a prominent lip, are greenish in colour, slightly whitish inside, and have no scent *[…]*. It is found in certain drier meadows of Pannonia and Austria *[…]*.

On the basis of the description and the woodcut, it is possible to attribute the plant to the genus *Epipactis*. The described morphological and ecological characteristics can be matched with certain species of the genus *Epipactis*. Of the possible attributions, *Epipactis helleborine* (L.) Crantz and *E. greuteri* H. Baumann & Künkele are the closest matches. However, the lack of detail in the historical description does not permit a species-level attribution. This entry illustrates the "descriptive threshold" of 16th-century botany: Clusius was able to correctly identify the characteristic floral morphology of the *Epipactis* group, but the minute colour and habitat differences that permit the differentiation of today's microspecies were not yet expressible in the standardized Latin terminology of his day.

### Elleborine III. (1583), Elleborine IIII. (1601)

There is a brief description of this species, but even this is enough to identify the plant high probability.

*[…] Similia interdum folia, nonnumquam multo latiora. Flores aut omnino purpurascentes, aut prope carnei. Secundi generis floribus prorsus similes. Nec radix differt.*

Caroli Clusii, *Rariorum plantarum historia* (Antverpiae, 1601), p. 273

*[…]* Its leaves are sometimes similar, sometimes much broader; the flowers are either entirely purplish or nearly flesh-colored, exactly like those of the second species [Elleborine II. 1601]; the root does not differ either.

The reddish-red flowers and the fact that the plant closely resembles *Cephalanthera longifolia* and/or *Cephalanthera damasonium* make it most likely to be *Cephalanthera rubra* (L.) Rich.. By framing this plant as a colour-variant of the same structural "type," Clusius demonstrates his "comparative-additive" method: he does not reinvent the description for each taxon, but focuses strictly on the diverging characters.

### Elleborine IIII. (1583), Elleborine V. (1601)

Clusius writes very briefly about this plant in both of his books, covering the entire text in these three sentences:

*Cubitali altitudine maius, amplis foliis. Oblonga purpurascentium florum spica nascitur in multis Pannoniae silvis, praesertim vero vbi Pseudodamasonium inueniri dixi.*

Caroli Clusii *Atrebatis Rariorum aliquot stirpium per Pannoniam, Austriam et vicinas quasdam provincias observatarum historia* (1583), p. 275.

Taller than a cubit, with large leaves. An oblong spike of purplish flowers arises in many forests of Pannonia, especially in those places where I previously stated that *Pseudodamasonium* (*Cypripedium calceolus*) occurs.

According to the above-mentioned characters, the plant can be most reasonably attributed to *Epipactis atrorubens* (Hoffm.) Besser. The tall stature, large leaves, and the oblong inflorescence of purplish flowers correspond well to this species. By particularly pointing out that this species grows in the same forest habitats as *Cypripedium calceolus*, Clusius shows an early insight into "site fidelity" and plant associations.

### Elleborine V. (1583), Elleborine VI. (1601)

**Fig. 9:**
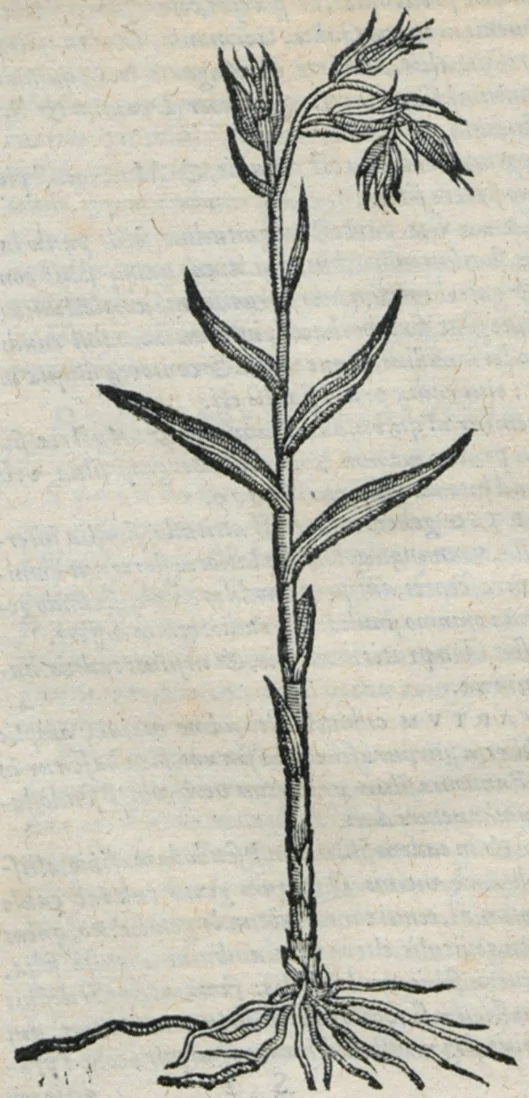
Elleborine V. Caroli *Clusii Atrebatis Rariorum aliquot stirpium per Pannoniam, Austriam et vicinas quasdam provincias observatarum historia* (1583), p. 276.

The plant is well described and included in both publications, along with illustrations (Fig. 9).

*[…]* *In singulis geniculis alternatim ambiunt angusta folia. Primi generis foliis non dissimilia. Summus caulis desinit in spicam florum sex, octo, aut plurium interdum, […] elegantissimo dilutoque purpurae colore. […] Labrum inferiore parte adnexum habens, aequalis fere cum foliis longitudinis, candicans, intus striatum. Sustinentur ii flores angulosa, contorta, atque nonnihil etiam hirsuta siliqua.* *[…]*.

Caroli Clusii *Atrebatis Rariorum aliquot stirpium per Pannoniam, Austriam et vicinas quasdam provincias observatarum historia* (1583), pp. 275–277.

*[…]* At each node, narrow leaves alternately clasp the stem. They are not dissimilar to the leaves of the first species. The stem terminates in a spike of six, eight, or sometimes more flowers, *[…]* of a very elegant, pale purplish colour. *[…]* The lip is attached at its lower part, nearly equal in length to the tepals, whitish, and striated within. These flowers are supported by angular, twisted, and even somewhat hairy capsules. *[…].*

As Clusius referred to Elleborine I. (1583), it is probably a *Cephalanthera* species, which is supported by the illustration as well. The red flowers with whitish-patterned lips make it clear that this is *Cephalanthera rubra*. Clusius’s systematic approach is evident in his cross-referencing of the leaves to those of "the first species" (Elleborine I.), indicating a deliberate attempt to build a comparative morphological framework.

### Elleborine VII. (1601)

This plant was only mentioned and described in the *Rariorum plantarum historia* (1601).

*[…] Erat vero illud pedalis fere altitudinis, gracili caule, tribus foliis prædito, Elleborines vulgaris foliis forma similibus, & quinque nervis per longitudinem eorum excurrentibus. Sed longe minoribus, ut quæ circiter unciam longa sint duntaxat, semunciam lata. Summus caulis in spicam florum desinit, Orchidis & Elleborines vulgaris flores imitantium, sed minorum. […].*

Caroli Clusii, *Rariorum plantarum historia* (Antverpiae, 1601), p. 273–274

*[…]* It was in fact approximately a foot in height, with a slender stem, furnished with three leaves similar in shape to those of the common Elleborine, and with five veins running along their length. But they were much smaller, being only about an inch long and half an inch wide. The upper part of the stem ends in a spike of flowers resembling those of Orchis and the common Elleborine, but smaller. *[…].*

Clusius described both the general habit and leaf morphology of the plant, which point to the genus *Epipactis*. The small stature, slender stem, and the presence of only three very small, narrow leaves distinguish it from the common Elleborine and are consistent with *Epipactis microphylla* (Ehrh.) Sw.. However, the limited detail of the description does not allow a fully confident species-level identification. While the historical description reaches its limit here in terms of floral detail, Clusius’s focus on the reduction of vegetative parts of the plant, highlights his ability to recognize "miniaturized" specialists within a genus.

### De Dentariis vvlgo dictis (1583) & Dentaria, vvlgo dicta (1601)

The group is based on the similarity of the rhizome of the plants classified here. The name Dentaria refers to the serrated rhizomes of the plants. According to current taxonomy, the group includes *Brassicaceae* species, and two orchids have also been identified.

**Fig. 10:**
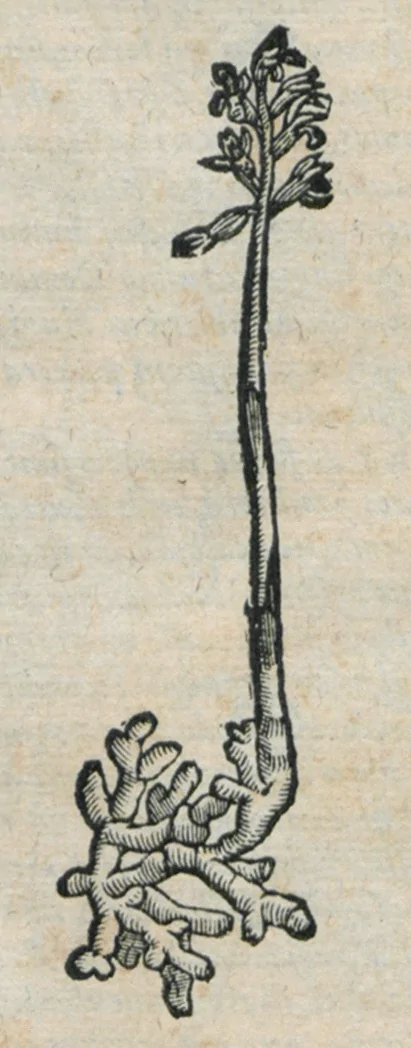
Dentaria coralloide radice III Caroli Clusii *Atrebatis Rariorum aliquot stirpium per Pannoniam, Austriam et vicinas quasdam provincias observatarum historia* (1583), p. 450.

The numbering in the two books is again inconsistent; Dentaria III. (1583) and Dentaria II. (1601) describe the same plant, and Dentaria IIII. (1583) and Dentaria III. (1601) also refer to the same plant. It is misleading that both works feature the same woodcut (Fig. 10) of an orchid with the number III., but in the books, Dentaria III. refers to a different species.

### Dentaria III. (1583) & Dentaria II. (1601)

Clusius correctly states in both works that they differ from the other members of the group, but classifies them here due to their similar rhizomes.

*Nihil habet commune cum superioribus tertia, praeter dentatam radicem. […] Cauliculum promit dodrantalem, aut paulo ampliorem. Succi plenum, & quasi transparentem, candicantem, sine foliis […]. Florem promit in summo binos aut ternos […], ex albo purpurascentes […]. Orchidis florum aemulos. Radix candida e multis veluti denticulis congesta est. Florentem Augusto mense invenimus ad radicem Etscheri iugi. […] Sub abietibus aliisque huius generis arboribus, quo sol vix unquam penetrat, nullaeque fere aliae stirpes nascuntur.*

Caroli Clusii Atrebatis Rariorum aliquot stirpium per Pannoniam, *Austriam et vicinas quasdam provincias observatarum historia* (1583), p. 449.

The third shares nothing in common with the preceding ones, except for the toothed root. *[…]* It produces a stem somewhat shorter than, a three-quarter foot or slightly exceeding, a foot in height, full of sap, almost translucent, whitish in colour, and without leaves *[…]*. At the summit it bears two or three flowers *[…]*, whitish turning purplish, *[…]* resembling the flowers of orchids. The root is white, composed as it were of many small tooth-like segments. *[…]* We found it in flower in the month of August at the foot of the Etsch^7^ range, beneath firs and other trees of this kind, where sunlight scarcely ever penetrates and almost no other plants grow.

Based on the described morphology, the plant can be confidently identified as the mycoheterotrophic orchid *Epipogium aphyllum* Sw.. The leafless, pale, translucent stem, the presence of only a few flowers, the toothed, coral-like root system, and the late flowering time in August are all characteristic of this species. Clusius’ account of the plant growing in deep shade beneath coniferous trees, in habitats with almost no other vegetation, further corresponds to the known ecology of *E. aphyllum*. He explicitly noted that its flowers were "mimicking those of orchids". This observation, combined with the precise ecological data, illustrates a methodological tension in early modern taxonomy.

### Dentaria IIII. (1583) & Dentaria III. (1601)

Both books contain descriptions of the plant, which may have been classified in the Dentaria group due to its peculiar rhizome.

*Quartae flores minores sunt, orchidis pumilae floribus pares […], ex herbaceo pallescentes. Labro tamen propendente exalbido, circa umbilicum purpureis radiis insignito. Inodori, quinque aut sex, spicae in modum in dodrantali caule enascentes. Qui foliis caret, & veluti subfuscis vaginulis reconditus est. Radicem habet ramosam corallii modo, omnino candidam. […] Hanc duntaxat observavi anno 1580 in silva recens caesa paulo supra Entzestorf.*

Caroli Clusii *Atrebatis Rariorum aliquot stirpium per Pannoniam, Austriam et vicinas quasdam provincias observatarum historia* (1583), p. 449

The flowers of the fourth species are smaller, comparable to those of a dwarf orchid *[…]*, and fade from a greenish colour. The lip, however, is protruding, pendent and whitish, adorned around the umbilical area with purple rays. They are scentless, with five or six flowers arising in a spike-like manner on a stem about three-quarters of a foot high. The stem bears no leaves and is, as it were, concealed within somewhat brownish sheaths. The root is branched, coral-like, and entirely white. *[…]* I observed this plant only once, in the year 1580, in a recently cleared forest somewhat above Entzestorf.^8^

The description of a leafless stem with brownish sheaths and a branched, white, coral-like root system allows for the confident identification of the mycoheterotrophic *Corallorhiza trifida* Châtel orchid. Clusius also provides an early phenological and land-use context. This establishes his work as a cornerstone of floristic documentation, capturing ephemeral, rare species that thrive in specific successional stages of forest habitats. This ecological niche further support the identification of *C. trifida.*

### Overall identification

Clusius wrote about a total of 19 orchid species (in modern terms) in his two publications (1583, 1601) (Table 1). Of these, 10 (52.63%) could be identified with the highest degree of certainty. 8 species (42.11%) described by Clusius were identified with a high degree of certainty, but without completely excluding other species. The most uncertain category includes 1 (5.26%) orchid described by Clusius, whose description fits several genera.

**Table 1:** Synopsis of the orchid identifications from Clusius' *Stirpium Historia* (1583) and *Rariorum plantarum historia* (1601)

Name used by clusius/designation	Modern scientific name (Taxon)
Orchis I.	Platanthera chlorantha (Custer) Rchb. ** or P. bifolia (L.) Rich. **
Orchis latifolia altera	*Orchis mascula* L.** *Orchis militaris* L.**
Orchis II.	*Gymnadenia* sp., * *Anacamptis* sp. * or *Orchis* s.l.*
Orchis III.	*Neotinea ustulata* (L.) R.M.Bateman et al., **
Orchis IIII. (Orchis Pannonica)	*Neotinea tridentata* ** (Scop.) R.M.Bateman et al., or *N.* × *dietrichiana* (Bogenh.) H.Kretzschmar **
Orchis V.	*Anacamptis morio* (L.) R.M.Bateman et al., ***
Orchis VI. (Orchis Batavica)	*Anacamptis pyramidalis (L.) Rich.* ***
Orchis VII. (1601)/VI. (1583)	*Herminium monorchis* (L.) R.Br. ***
Orchis VIII. (1601)/VII. (1583)	*Dactylorhiza sambucina* (L.) Soó ***
Pſeudoorchis	*Asparagaceae s.l*. (not orchid) *
Pſeudo-leimodωron/Limodoro Austriaco	*Limodorum abortivum* (L.) Sw. ***
Psevdodamasonio (1583)	*Cypripedium calceolus* L. ***
Elleborine I. (1583)/II. (1601)	*Cephalanthera damasonium* (Mill.) Druce ** or *C. longifolia* (L.) Fritsch **
Elleborine II. (1583)/III. (1601)	*Epipactis helleborine* (L.) Crantz** or *E. greuteri* H.Baumann & Künkele **
Elleborine III. (1583)/IIII. (1601)	*Cephalanthera rubra* (L.) Rich.***
Elleborine IIII. (1583)/V. (1601)	*Epipactis atrorubens* (Hoffm.) Besser**
Elleborine V. (1583)/VI. (1601)	*Cephalanthera rubra* (L.) Rich.***
Elleborine VII. (1601)	*Epipactis microphylla* (Ehrh.) Sw.**
Dentaria III. (1583)/II. (1601)	*Epipogium aphyllum* Sw. ***
Dentaria IIII. (1583)**/**III. (1601)	*Corallorhiza trifida* Châtel. ***

***Definitive Identification: Morphological description and illustration, together with phenological data, may enable identification beyond doubt

**Species-level Approximation: The description and/or illustration clearly indicates a particular species or a narrow species complex, but inherent morphological variability or shared characters with closely related taxa, preclude absolute certainty

*Genus-level or Uncertain Identification: The information provided is not sufficient for identification at the species level; the description refers to general characteristics that could apply to several different species or different genera

## Discussion

The analysis of Clusius’s orchid corpus allows us to revisit the broader question of species ontology in early modern botany. This study does not claim to be a comprehensive historical study of pre-Linnaean classification; instead, it offers an interdisciplinary reassessment of Clusius’s orchid corpus as a tool for evaluating the structural coherence of descriptive practice across epistemic regimes. Any attempt to interpret sixteenth-century botanical sources through the baseline of modern Linnaean taxonomy inevitably entails a degree of anachronism (Szabó, 2005). To address this, the present study avoids a teleological reading of history by employing modern taxonomy strictly as a scientific control group. Retrospective agreement with modern taxonomy is less a proof of ontological equivalence than an indication of structural compatibility between observational practices across epistemic regimes. Within this framework, the identification of orchids serves as an epistemological probe to evaluate the correspondence between Clusius’s empirical observations and objective biological entities. By using modern taxonomy as a benchmark, it becomes evident that Clusius did not simply discard utilitarian classifications, but instead built upon and expanded the empirical observational practices established by his predecessors such as Leonhart Fuchs (1501–1566) and Valerius Cordus (1515–1544). His work represents a highly developed form of scholarly folk taxonomy, where intuitive, gestalt-based recognition of natural kinds—rooted in a structural understanding of morphological stability—was refined into a rigorous discursive framework (Atran, 1990; Berlin, 1992). This approach reflects an internal evolution of natural history, transitioning from a focus on medicinal virtues toward a broader, systematic documentation of natural diversity as a characteristic practice of the era.

Carolus Clusius was the central figure of this transition in Europe, maintaining extensive scientific and cultural networks, including close connections with Hungarian humanists (Orbán, 2023). The Pannonian flora provided the impetus for this change; the region’s diverse flora compelled Clusius to combine a critical analysis of ancient botanical literature with direct field observations (Egmond, 2010). His work is characterized by the tension between classical philology and emerging empiricism. Yet, Clusius stopped short of classifying all orchids into a single group. He often followed 16th-century classification practices based on leaves and consistently noted that these plants possess distinctive reproductive characteristics, such as orchid-like flowers or dust-like seeds. This indicates an highly precise empirical understanding of the *Orchidaceae* family as a natural group, even before the concept of a family officially existed (Gombocz, 1936/2007). As the first to provide a comprehensive account of the Pannonian flora, Clusius’s documentation is of importance; it represents the earliest scientific baseline for the regional history of orchids and the development of modern floristics (Gombocz, 1936/2007; Molnár, 2011). In Clusius's works, morphology functions as a descriptive tool, while also acting as an ontological indicator. Persistent structural characteristics, especially reproductive traits, serve as indicators of natural continuity between individual variations. Clusius’s botanical practice is characterized by a systematic integration of ecological and phenological observations. While the inclusion of locus (place) and tempus (time) was part of the broader botanical tradition, Clusius expanded this methodological scope by synthesizing these elements with detailed observations of regional rarity and specific environmental conditions, thereby refining the empirical standards of his time.

### Clusius and his contemporaries

Clusius’s description of orchids—compared to the works of Leonhart Fuchs (*De historia stirpium*, 1542) and Matthias de l’Obel (1538–1616) (*Plantarum seu stirpium historia,* 1567)—show similar characteristics. In contrast, however, Clusius reveals new methodological technique when compared with the two other authors. Though all three authors moved beyond a strictly medicinal focus and included detailed botanical descriptions in their works, their priorities differed widely. Fuchs relied largely on classical literature (Dioscorides, Galen, Pliny) and was interested in the medicinal uses of plants. Meanwhile, Clusius placed greater emphasis on the ecological context and the precise morphology of the plants’ reproductive organs. In particular, Clusius demonstrated a consistent ability to recognize natural groupings; he frequently noted when various taxa shared 'orchid-like' traits, demonstrating a recognition of morphological stability that would later become a cornerstone of formal classification. On the contrary, such an approach is characteristic of only one instance in Fuchs's book, where he mentions similarities of Satyrium (a *Dactylorhiza* species) with Cynosorchis by leaves. Pietro Andrea Mattioli (1501–1578) lacked this diagnostic focus on generative and vegetative characters. Clusius’s reliance on generative structures, particularly the 'dust-like' seeds, clearly shows his commitment to empirical observation as a primary source of botanical knowledge. Furthermore, while Mattioli utilized traditional nomenclature rooted in classical literature, Clusius’s use of numerical indexing suggests a shift toward a practical, field-based system of identification, underscoring his reliance on direct experience over classical citations.

This methodological focus on regional particularity and descriptive accuracy is further illustrated by the case of Matthias de l’Obel (1538–1616); while he documented several *Ophrys* species, Clusius’s emphasis on Pannonian flora resulted in a much more subtle documentation of the Elleborine group (*Epipactis*, *Cephalanthera*), which were commonly treated only superficially by his contemporaries (Teoh, 2019). The contrast between specialized botanical inquiry and traditional practical herbalism is also evident in the example of András Beythe (1564–1599); despite Clusius’s direct influence, Beythe’s medicinal herbal (1595) completely excluded orchids, likely due to their lack of known pharmacological significance in the local Hungarian tradition (Beythe, 1595; Gombocz, 1936/2007).

Ultimately, Clusius’s botanical practice is best understood as an exceptionally rigorous application of observational standards shared by contemporaries like Rembert Dodoens (1517–1587). Comparing Clusius’s approach to Dodoens’s *Stirpium historiae pemptades sex* (1583) reveals that both authors operated within a framework where the study of plants for their own sake was already gaining momentum. As Moran (2017) argues, these botanical descriptions were products of 'structures of practical knowledge,' reflecting a complex integration of observation and artisanal skill. Thus, Clusius’s work can be understood as a development within the scholarly tradition of herbalism, which increasingly treated morphological traits as diagnostic units, broadening the basis for what would later be formalized as scientific taxonomy.

This comparative analysis shows that Clusius and his contemporaries did not classify plants merely for utilitarian purposes. By consistently relying on stable morphological and ecological characteristics—which extended beyond local or medicinal contexts—they proceeded from the implicit assumption that species are permanent, natural entities. Their work thus suggests that early modern botanical categories were more than simple linguistic or utilitarian constructs; they were rooted in the recognition of stable, objectively observable natural units.

### Visual evidence and the materiality of botanical illustration

In the absence of surviving sixteenth-century herbarium specimens for many of these taxa, botanical illustrations provide essential visual context for interpreting Clusius's descriptive observations. As Pietro Antonio Michiel (1510–1576) maintained in the mid-sixteenth century, naturalistic and detailed illustrations are indispensable supplements to textual descriptions (Egmond, 2010). Clusius' works are suitable examples to show how the synergy between text and image could be highly sophisticated. Instead of treating these illustrations as passive, specimen-like records, we can see these illustrations—much like the practical knowledge structures discussed by Moran (2017)—as products of an embodied dialogue between natural observation and printmaking craftsmanship.

Furthermore, the material economy of these woodblocks—which often circulated across various printing houses—cautions against treating them as fixed representations unique to a single author (Chen, 2020). The illustration of Elleborine II. (1583)/III. (1601) (Fig. 8) serves as a compelling bibliographical 'time-stamp' regarding the production history of the *Rariorum plantarum historia* (1601). A comparative analysis of various digitized copies reveals that the woodblock underwent physical degradation during the printing process. While the copy held at the Missouri Botanical Garden preserves the woodblock in its intact state, most extant copies—including those from the National Library of Naples and the Austrian National Library—exhibit a characteristic breakage of the bottom leaf. This damage appears consistently in later works, such as Rembert Dodoens’s *Cruydt-boeck* (1608) (p. 682), while the intact woodblock is found in earlier publications, such as Matthias de l’Obel’s *Plantarum seu stirpium historia* (1576) (p.169) and *Icones stirpium* (1591) (p.312). Although the absence of a single leaf fragment—considering the plant's habit—does not necessarily render taxonomic identification impossible, the physical degradation of the woodcut nevertheless diminished the epistemological weight of the visual source: for the early modern botanist, the authenticity of the botanical illustration functioned as a surrogate for *autopsia* (direct visual inspection), meaning that any technical imperfection inevitably eroded the diagnostic certainty of the description (Kusukawa, 2012).

These material 'traces' confirm that botanical illustrations were not static, uniform prints, but evolved through the iterative, often fragmented, economy of the early modern book trade. Consequently, these woodcuts functioned both as identification aids for the educated public and as aesthetic elements that may have contributed to the commercial and scientific value of botanical works. This versatility aligns with the broader role played by images in early modern book publishing, as discussed by Pettegree and der Weduwen (2019).

### Epistemological shifts: integrative description versus diagnostic minimalism

By comparing Clusius’s descriptions with modern taxonomic requirements, a marked shift in the epistemology of descriptive practice can be observed. To ensure stability in the use of names, modern practice gives priority to standardized diagnostic characteristics, which often focus on isolated reproductive morphology. Clusius’s descriptions, however, reflect a more holistic approach to the organism. To illustrate, where a modern diagnosis might reduce a taxon to its definitive floral traits, Clusius’s accounts integrate phenological stages, sensory data, and site-specific ecological observations. Transcending a mere historical curiosity, this contrast holds significant relevance for modern science, as Clusius’s methodological stance illustrates that 'species ontology' is shaped by ecological plasticity as much as by morphological boundaries. By documenting elements as scent, habitat, and reproductive morphology alongside visible appearance, Clusius preserved a broad range of biological information that could contribute to more precise plant identification. Consequently, the reassessment of these early modern descriptions invites current-day botanists to reconsider the utility of integrative descriptions, suggesting that the integration of environmental and phenological data, which was central to Clusius, provides a more comprehensive framework for understanding species stability than the current emphasis on diagnostic minimalism.

### Evaluating taxonomic correspondence: a tripartite analysis

Analysis of the correspondence between Clusius’s pre-Linnaean categories and modern nomenclature shows a complex observation pattern. The correlation between early modern observations and contemporary taxa is far from uniform; rather, it falls into three distinct categories, reflecting the dynamic between Clusius’s observation accuracy and the biological complexity of the *Orchidaceae*.

### High-confidence identifications: empirical stability across centuries

A significant portion of Clusius’s orchid corpus exhibits such descriptive stability that identification remains unequivocal even by modern standards. These cases provide strong example of Renaissance botanical observation, where key diagnostic traits—often omitted by his contemporaries—were correctly identified as stable markers. The high degree of correspondence between Clusius’s records and modern taxa suggests that detailed empirical observation could produce categories that remain compatible with later biological classifications.

*Anacamptis morio* (Orchis V.): The detailed documentation of extreme colour polymorphism (purple, flesh-coloured, white) and the "blunt spur" provides a definitive link to this species, a conclusion consistent with Gombocz (1936/2007).

*Anacamptis pyramidalis* (Orchis VI. 1601): Despite the mention of an atypical odor, the woodcut’s depiction of a compact, pyramidal inflorescence and three-lobed labellum strongly supports this identification, a view echoed by Teoh (2019).

*Herminium monorchis* (Orchis VI 1583/VII. 1601): The "musky scent" and the unique "single round tuber" (*unicam radicem, rotundam*) are diagnostic features that Clusius recorded with precise accuracy, allowing for no other plausible interpretation when compared to modern floristic data (Molnár, 2011).

*Dactylorhiza sambucina* (Orchis VII. 1583/VIII. 1601): Clusius’s specific comparison of the scent to "elderflowers" remains one of the earliest and most accurate diagnostic uses of fragrance in orchidology, an identification supported by both Gombocz (1936/2007) and Teoh (2019).

*Cephalanthera rubra* (Elleborine V. 1583/VI. 1601): This taxon can be identified with high certainty based on the dark red flower colour and whitish pattern on the labellum described by Clusius. Clusius' precision is demonstrated by the fact that he directly compared the plant's habit (structure) with other *Cephalanthera* species he also described, creating a clear morphological continuity within the group. The characteristics described are fully consistent with modern identification criteria (Kühn et al., 2019). The fact that Elleborine III. (1583)/IIII. (1601) is probably the same species, just a different population (and therefore may differ in phenotype) supports Clusius's great precision and accurate observations.

*Limodorum abortivum* (De Limodoro Austriaco/Leimodoron): Clusius' description of *Limodorum* is precise; he correctly recognized the orchid-like structure of the flowers and the plant's unusual habit resulting from its lack of chlorophyll. While the 1583 record relied on direct observation, the 1601 *Historia* added critical comparisons with ancient authorities, making this one of his most stable identifications with the modern flora work (Kühn et al., 2019; Molnár, 2011). It is especially important that Clusius did not pass over in silence those specimens that differed from the common ones: he noted a considerably paler one in the vicinity of Montpellier. This observation allows for two interpretations that are exciting from a scientific history perspective. On the one hand, it may reflect the phenotypic variability resulting from the wide geographical distribution of *L. abortivum*. On the other hand, the description may also reflect an early recognition of *Limodorum trabutianum* Batt. (a Mediterranean species), suggesting a notable degree of ecological awareness in Clusius’s observations. Clusius's remark that he observed the pale form in oak forests (while *L. abortivum* occurs predominantly in pine forests) coincides precisely with the modern ecological separation of the two species (Kühn et al., 2019). Although Clusius did not formally separate the two taxa, his recording of subtle differences testifies to his early empirical recognition of intraspecific variation and ecological differentiation.

*Cypripedium calceolus* (Psevdodamasonio): It represents a notable instance of Clusian botany and a significant advancement in the history of plant representation. Clusius’s illustration is notable for its time, as it depicts both vegetative and generative organs in meticulous detail. Unlike earlier representations by Dodoens and Lobelius, which relied on woodcuts lacking fruit structures, Clusius included the capsules and seeds (Cribb, 2014). This inclusion suggests that he associated the species with orchids not merely through floral mimicry, but by recognizing its reproductive biology and characteristic "dust-like" seeds. Clusius’s empirical "eye" also captured intraspecific phenotypic variability, such as rare white-flowered individuals, and recorded the vernacular name "Marien schuch", highlighting the species' cultural salience in local knowledge systems. From a philological perspective, the slight orthographic variation, appearing as *Pſeudodamaſonium* in the *Stirpium nomenclator Pannonicus* (1583) and *Psevdodamasonio* in the descriptive *Rariorum aliquot stirpium…* (1583), reflects the lack of orthographic standardization in 16th-century Latin rather than a taxonomic shift (Ogilvie, 2006). The functional difference between a nomenclatural list and a descriptive treatise explains why earlier studies (Szabó, 1992) found the taxon difficult to identify. But by combining the characteristics and woodcut from the 1601 version, the true identity of the taxon can be verified as being *Cypripedium calceolus*.

*Corallorhiza trifida* and *Epipogium aphyllum* (Dentaria group): Both of them considered certain based on modern morphological and biogeographical standards (Kühn et al., 2019; Molnár, 2011). Clusius’s record of *E. aphyllum* in deep shade confirms that habitat was treated as a relevant component of botanical description. While the physiological concept of mycoheterotrophy was centuries away from being defined, Clusius’s empirical record of its specific habitat is one of the earliest documented indications of this ecological niche.

### New findings: re-evaluating historical hypotheses

On the basis of re-analysis of Latin sources against modern floristic databases, a number of identifications have been obtained, which correct or supersede the interpretation of 20th-century literature. These findings underline the importance of joint analysis of descriptive and ecological information for the taxonomic resolution of pre-Linnaean sources.

*Platanthera chlorantha* (Orchis I.): While Gombocz (1936/2007) tentatively suggested *Orchis purpurea*, the present analysis emphasizes the diagnostic weight of Clusius' morphological remarks. Clusius highlighted the "elongated twin tubers" and the unusually broad leaves. These characters, combined with the *Platanthera* genus, suggest *Platanthera chlorantha* as the most likely match based on morphological features and biogeographical reality, according to Lorenz et al., (2025), but I believe that *P. bifolia* cannot be completely ruled out based on the description.

*Orchis mascula* (Orchis latifolia altera): The woodcut has a decisive role, since the description alone applies to several taxa. The dorsal petal and the open dome created by the petals fit well with the characteristics of *Orchis mascula*, whereas the floral structure of *O. purpurea* usually shows a rather compact construction of the inflorescence (Kühn et al., 2019). In his work (1936/2007), Gombocz classifies both *O. militaris* and *O. purpurea* as belonging to this species. The reuse of the same illustration in the works of Lobelius, where it was likewise identified as *O. mascula* (Teoh, 2019), further supports this interpretation.

*Epipactis helleborine* (Elleborine II. 1583/III. 1601): Clusius' morphological description does not completely rule out the presence of *Epipactis greuteri* in terms of certain characteristics (e.g., based on the habit and characteristics of the flowers). Although identification is statistically less likely due to the rarity of the latter species, the possibility remains, indicating the depth of Clusius' observations, which may have even included such rare taxa. Gombocz (1936/2007) associated these descriptions with *Epipactis palustris*^9^ (L.) Crantz, but Clusius clearly stated that he observed the plants in dry habitats. Since *E. palustris* is strictly associated with wet habitats (marshes, swamp meadows), the highly variable *Epipactis helleborine* seems to be the most likely candidate, which is also common in the drier parts of the Pannonian forests described by Clusius (Kühn et al., 2019; Molnár, 2011). This point highlights the primary filtering role of ecological data in historical identification, while also marking the limits of descriptive prose.

*Epipactis atrorubens* (Elleborine IIII. 1583/V. 1601): Gombócz (1936/2007) identified the plant as *Epipactis helleborine*.^10^ Though this identification can be well explained on the basis of leaf size, the uniform red-violet colour as described by Clusius is not typical of *E. helleborine*. Rather, these features are much more typical of *Epipactis atrorubens*, which always has dark reddish-purple flowers. Clusius also pointed out some differences in colour and habit that were often neglected by later botanists. Furthermore, his explicit mention of shared habitats between *E. atrorubens and C. calceolus* indicates that his taxonomic intuition was informed not only by morphology but by a primitive understanding of synecology. He was recognizing that certain orchids act as ecological indicators for one another long before the formalization of community ecology.

### Phenotypic plasticity and hybridization: the challenges of pre-linnaean documentation

Not all identifications are equally secure, revealing the inherent limits of retrospective stabilization. Far from representing a failure of sixteenth-century empiricism, this ambiguity reflects a faithful recording of the biological complexity and phenotypic plasticity inherent in certain orchid groups. Observations of such variability, which are interpreted in modern biology as phenotypic plasticity, demonstrate that Clusius’s work often reflects the natural variation of populations before the artificial "fixity" imposed by later nomenclatural types. Clusius’s careful documentation of colour variation, floral polymorphism, and ecological differences indicates that species were not treated as rigid typological boxes. Instead, variation was recorded as part of a broader empirical reality, suggesting a flexible yet stable conception of natural kinds grounded in recurring morphological structure.

*Neotinea* Complex (Orchis III. & IIII.): The identification of Orchis III. and Orchis IIII. reveals the challenges of documenting the *Neotinea* genus in the Pannonian region. While Orchis III. aligns with *Neotinea ustulata*, the description of Orchis IIII. (purple helmet, specific fragrance) points strongly toward *Neotinea tridentata*. However, the accompanying woodcut shows the presence of intermediate individuals. As the two species are known to form natural hybrids (*Neotinea* × *dietrichiana*) in their shared natural ranges (Djordjević et al., 2012), the fact that Clusius’s observation was “ambiguous” is most likely due to the fact that it was the first recorded instance of a hybrid swarm or a very plastic population.

The Mystery of Orchis II.: This taxon remains one of the most enigmatic entries. Clusius describes a plant from the Grebén ridge with "narrow leaves" and "beautiful red flowers". Gombocz (1936/2007) proposed *Dactylorhiza incarnata*,^11^ nevertheless, the personal categorization of orchids by Clusius into those with rounded versus finger-shaped tubers strongly rejects this proposal, as he assigned Orchis II. to the rounded-tuber category. This difference implies that Clusius possibly encountered a rare local phenotype or a rare population of the species *Gymnadenia* or *Anacamptis* with unusual flower coloration. The aspect that this form does not match any present day "standard" specimen emphasizes that old literature can retain information about former botanical diversity or rare phenotypes that are no longer found in those particular regions.

Phenotypic Plasticity in the Elleborine Group: The internal structure of the Elleborine group faithfully reflects the methodological constraints of 16th-century botany and Clusius's thoroughness as an observer. During this period, classification was primarily based on leaf morphology, with taxa often distinguished on the basis of a single dominant characteristic (Cambiaire, 2023). From today's perspective, Clusius's Elleborine category includes two separate genera, *Cephalanthera* and *Epipactis*, but his descriptions mostly allow for their precise separation. Although the identification of the white-flowered *Cephalanthera* (Elleborine I. 1583/II. 1601) is uncertain between *C. damasonium* and *C. longifolia*, Clusius' explicit comparison with the leaves of *C. rubra* shifts the balance towards the broader-leaved *Cephalanthera damasonium*.

Morphological Boundaries and Small-Statured Taxa: The case of Elleborine VII. (1601) exemplifies Clusius's powers of observation and precision. While the characteristics of the small habit, slender stem, and three narrow leaves bear a striking resemblance to the modern concept of *Epipactis microphylla* (Kühn et al., 2019; Molnár, 2011), the dryness of 16th-century writing makes it impossible to assign a species-level identification. The relevance of this entry is mainly to illustrate the diagnostic acumen of Clusius, who was able to distinguish a small morphological form from the taller Elleborine species, despite the limitations of past naming conventions.

### Pſeudoorchis: methodological challenges of morphological analogy

The mention of “Pſeudoorchis” (1601) in the chapter on Orchids is one of the most prominent examples of Clusius’s taxonomic thinking and clarity. With regard to this taxon, Clusius demonstrates a critical perspective toward his own methodological approach, emphasizing the challenges inherent in botanical classification within the epistemological context of the sixteenth century. The plant’s classification as an orchid was based on a series of morphological characteristics. Although he recognized that the membranous bulbous root system contradicted the nature of orchids, he assumed a kind of "transitional nature" due to the similarity of the inflorescence remnants and the capsules.

### Methodological reflections and synthesis: from *materia medica* to empirical botany

Recent scholarship has complicated the view that species stability emerged only with Linnaean formalization. Ogilvie (2006) documented the Renaissance shift toward empirical morphology, while Reeds (1976) demonstrated that humanist botanists sought referential stability beneath proliferating plant names. Minelli (2019) further argues that species recognition was sustained through diverse historical naming practices, independent of binomial nomenclature. Against this backdrop, Clusius’s treatment of orchids—morphologically complex and ecologically specialized plants—offers a stringent test of early modern biological recognition. The high degree of correspondence found here between Clusius’s descriptions and modern taxonomic units supports the view that his botanical practice extended beyond the primarily utilitarian perspective of Renaissance herbalism. While his contemporaries often prioritized pharmacological virtues, Clusius’s 'Pannonian experience' fostered an empirical approach that contributed significantly to the development of rigorous floristic documentation. Although the absence of extant herbarium specimens requires a 'triangulation' of Latin prose, woodcut iconography, and biogeographical data, the evidence suggests an orientation toward stable biological entities. By capturing phenotypic plasticity and reproductive morphology—features like dust-like seeds and capsule forms—Clusius grasped the biological truth of the living plant rather than a fixed, idealized type. Finally, his approach shows a clear effort to circumscribe regular morphological series that correspond very well to our contemporary species definitions. Clusius’s Pannonian botanical activity therefore provides quite an early empirical baseline for the subsequent study of the region’s orchid diversity, bridging humanist botanical description and later natural history.

## Conclusion

Carolus Clusius’s documentation of orchids in the sixteenth century stands as a testimony to the power of direct observation over inherited authority. Through the systematic re-evaluation of his Pannonian works, it can be concluded that his descriptions were directed toward objective biological entities, many of which remain identifiable with high confidence today. Clusius consistently integrated detailed morphological observations with ecological setting, phenology, and comparative examination of earlier authors. His treatment of orchids demonstrates an early understanding of natural variation, habitat characteristics, and reproductive traits that anticipates key elements of modern systematic thinking. The few instances of taxonomic ambiguity in his writings are thus not indicative of a lack of precision but instead represent the accurate transmission of the natural complexity and plasticity of the *Orchidaceae* by Clusius. His legacy extends beyond questions of history and philology; his work represents an important stage in the development of botanical science, marking a transition from the study of medicinal plants toward field observation and from description toward a more dynamic understanding of nature. The orchids, with their morphological complexity and ecological particularity, represented an epistemological stress test for botany in the early modern period. The accuracy of Clusius’s observations suggests that the development of taxonomy involved the codification of observational practices that were already capable of recognizing biological form in nature.

## Footnotes

1 In Clusius’ Pannonian works, “Pannonia” refers not to the Roman province sensu stricto, but rather denotes a broader historical, geographical region centred on western and northwestern territories of the Kingdom of Hungary, as well as adjacent parts of the Archduchy of Austria. In the sixteenth century, this area formed a frontier zone of the Habsburg Monarchy. Clusius treated Pannonia primarily as a natural landscape unit, largely independent of political borders. Consequently, his “Pannonian” localities are today distributed mainly across Hungary, eastern Austria (Burgenland and Lower Austria), Slovakia, and marginally Slovenia and Croatia.

2 the use of 'v' for 'u', 'j' for 'i', 'ſ' for 's', and the terminal ‘J’ in Roman numerals.

3 historical place name used by Clusius for the settlement known today as Güssing (Burgenland, Austria). At the time of Clusius’ observations, the town (Németújvár in Hungarian) formed part of the Kingdom of Hungary; it has belonged to Austria since 1921.

4 The original Roman numerals (e.g. “IIII.”) are retained following Clusius’ usage in the original editions.

5 The term “genus” is used here in the sense applied by Clusius, referring to a morphologically defined group rather than to a taxonomic genus in the modern Linnaean sense.

6 During Clusius's time, it belonged to the Kingdom of France, and the area retained its name; currently Montpellier, France.

7 historical name of the Adige River, referring to the Adige valley in northern Italy, including parts of present-day South Tyrol. In modern geographical terminology, this area corresponds to the Val d’Adige (Adige Valley).

8 corresponds to Enzesdorf an der Fischa or its surroundings near Enzesfeld in present-day Lower Austria. At the time of Clusius’ observations, this area formed part of the Kingdom of Hungary within the Habsburg territories, whereas today it belongs to Austria.

9 The species is found in Gombocz's (1936/2007, p. 97) work under the name *Helleborine palustris* (L.) SCHRK.

10 The species is found in Gombocz's (1936/2007, p. 97) work under the name *Helleborine latifolia* (L.) DRUCE.

11 The species is found in Gombocz's (1936/2007, p. 102) work under the name *Orchis incarnata* L.
